# DNA damage contributes to neurotoxic inflammation in Aicardi-Goutières syndrome astrocytes

**DOI:** 10.1084/jem.20211121

**Published:** 2022-03-09

**Authors:** Anna Maria Sole Giordano, Marco Luciani, Francesca Gatto, Monah Abou Alezz, Chiara Beghè, Lucrezia Della Volpe, Alessandro Migliara, Sara Valsoni, Marco Genua, Monika Dzieciatkowska, Giacomo Frati, Julie Tahraoui-Bories, Silvia Clara Giliani, Simona Orcesi, Elisa Fazzi, Renato Ostuni, Angelo D’Alessandro, Raffaella Di Micco, Ivan Merelli, Angelo Lombardo, Martin A.M. Reijns, Natalia Gromak, Angela Gritti, Anna Kajaste-Rudnitski

**Affiliations:** 1 San Raffaele Telethon Institute for Gene Therapy, Istituto di Ricovero e Cura a Carattere Scientifico San Raffaele Scientific Institute, Milan, Italy; 2 Vita-Salute San Raffaele University, School of Medicine, Milan, Italy; 3 Sir William Dunn School of Pathology, University of Oxford, Oxford, UK; 4 Department of Biochemistry and Molecular Genetics, University of Colorado Anschutz Medical Campus, Aurora, CO; 5 Department of Molecular and Translational Medicine, “Angelo Nocivelli” Institute for Molecular Medicine, University of Brescia, Azienda Socio Sanitaria Territoriale Spedali Civili, Brescia, Italy; 6 Department of Brain and Behavioral Sciences, University of Pavia, Pavia, Italy; 7 Child Neurology and Psychiatry Unit, Istituto di Ricovero e Cura a Carattere Scientifico Mondino Foundation, Pavia, Italy; 8 Unit of Child Neurology and Psychiatry, Brescia, Department of Clinical and Experimental Sciences, University of Brescia, Azienda Socio Sanitaria Territoriale Spedali Civili, Brescia, Italy; 9 Medical Research Council Human Genetics Unit, Institute of Genetics and Cancer, The University of Edinburgh, Edinburgh, UK

## Abstract

Aberrant induction of type I IFN is a hallmark of the inherited encephalopathy Aicardi-Goutières syndrome (AGS), but the mechanisms triggering disease in the human central nervous system (CNS) remain elusive. Here, we generated human models of AGS using genetically modified and patient-derived pluripotent stem cells harboring *TREX1* or *RNASEH2B* loss-of-function alleles. Genome-wide transcriptomic analysis reveals that spontaneous proinflammatory activation in AGS astrocytes initiates signaling cascades impacting multiple CNS cell subsets analyzed at the single-cell level. We identify accumulating DNA damage, with elevated R-loop and micronuclei formation, as a driver of STING- and NLRP3-related inflammatory responses leading to the secretion of neurotoxic mediators. Importantly, pharmacological inhibition of proapoptotic or inflammatory cascades in AGS astrocytes prevents neurotoxicity without apparent impact on their increased type I IFN responses. Together, our work identifies DNA damage as a major driver of neurotoxic inflammation in AGS astrocytes, suggests a role for AGS gene products in R-loop homeostasis, and identifies common denominators of disease that can be targeted to prevent astrocyte-mediated neurotoxicity in AGS.

## Teaser

Giordano et al. establish human AGS iPSC-based models of *TREX1* or *RNASEH2B* deficiencies. They uncover DNA damage as a major driver of neurotoxic inflammation in AGS astrocytes that can be uncoupled from aberrant type I IFN responses and identify novel targets to curtail AGS neuroinflammation.

## Graphical Abstract

**Figure:**
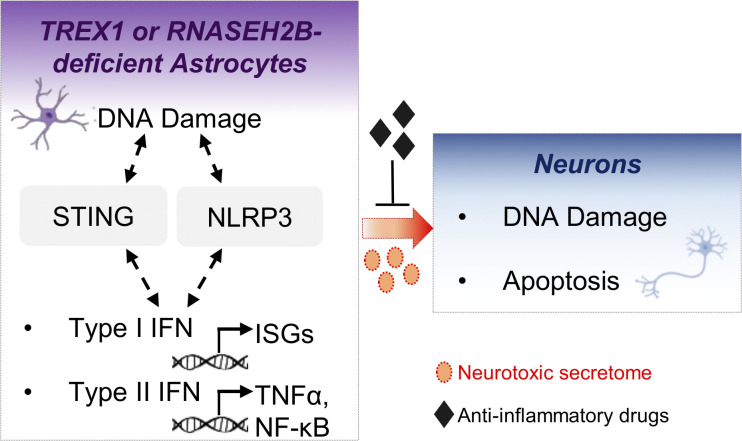

## Introduction

Aicardi-Goutières syndrome (AGS) is a monogenic inflammatory encephalopathy caused by mutations in any one of nine genes (*TREX1*, *RNASEH2A/B/C*, *SAMHD1*, *ADAR1*, *IFIH1*, *LSM11*, and *RNU7-1*) encoding for proteins involved in the metabolism and detection of nucleic acids (NAs; Aicardi and Goutieres, 1984; Crow et al., 2015b; Uggenti et al., 2020). Aberrant activation of cell-intrinsic pathways of NA sensing is emerging as a potential driver of several autoinflammatory and autoimmune diseases (Crow and Manel, 2015). Among these, aberrant sensing of NAs originating from the expression of endogenous retroelements (EREs) or accumulating DNA damage and the consequent increase in type I IFN levels have been suggested to be primary drivers of AGS pathogenesis (Barrat et al., 2016; Lee et al., 2019; Potenski et al., 2019; Volkman and Stetson, 2014). However, most mechanistic studies investigating the roles of AGS proteins in NA metabolism used non-neural cell lines or mouse models, most of which fail to replicate the central nervous system (CNS)–related AGS symptoms and are therefore not well suited to study the complexity of AGS neuropathology.

*TREX1* was the first gene to be associated with AGS (Crow et al., 2006a). It encodes a DNase able to degrade both single- and double-stranded DNA (ssDNA and dsDNA) in a 3′- to -5′ fashion (Grieves et al., 2015; Mazur and Perrino, 1999). Mutations in this enzyme can result in the accumulation of intracellular DNA able to trigger type I IFN responses (Stetson et al., 2008a; Yang et al., 2007). Besides activating type I IFN responses, TREX1 deficiency leads to inflammatory myocarditis, activation of DNA damage, and autoimmune responses in mice (Morita et al., 2004; Yang et al., 2007). The most frequent cause of AGS is mutations in *RNASEH2B*, which encodes one of the three subunits of the RNase H2 complex. Almost half of AGS patients have biallelic mutation in one of the three RNase H2 subunits (Crow, 2015; Crow et al., 2006b), with the A177T mutation in *RNASEH2B* being the most common (Crow et al., 2015b). RNase H2 is an essential enzyme that cleaves the RNA strand of RNA/DNA heteroduplexes as well as single embedded ribonucleotides in dsDNA (Cerritelli and Crouch, 2009; Reijns and Jackson, 2014). The latter function is widely studied because it is the first step of ribonucleotide excision repair (Sparks et al., 2012), the process essential for removal of 1 million ribonucleotides misincorporated in mammalian cells during DNA replication (Reijns et al., 2012; Uehara et al., 2018). Much less is known about RNase H2 cleavage of cellular RNA/DNA hybrids, such as those found in R-loops, despite RNase H2 being the main cellular source of such RNase H activity in the nucleus (Busen, 1980; Reijns et al., 2012). Notably, increased RNA/DNA hybrid formation has previously been reported in AGS patient–derived fibroblasts (Lim et al., 2015), but the role of R-loops in cells of the human CNS and across AGS gene defects remains unexplored.

Induced pluripotent stem cells (iPSCs) are attractive tools for disease modeling, particularly when the affected tissue is not available for cell purification, e.g., the CNS, and studying which aspects of cell development are crucial for pathogenesis (Marchetto and Gage, 2012). Human iPSCs have recently been exploited to model TREX1 deficiency, providing evidence for astrocyte contribution to AGS pathology (Thomas et al., 2017). In this context, treatment with reverse transcriptase inhibitors was shown to block accumulation of LINE-1–derived NAs and to rescue associated toxicity in TREX1-deficient neural progenitor cells. Although EREs are among the most investigated sources of pathological immune activation in AGS to date, other NA sources may also contribute. Indeed, inhibition of reverse transcriptase (RT) in TREX1-deficient AGS patients gave some degree of benefit in some but not all patients in a recent clinical trial (Rice et al., 2018). Furthermore, TREX1-deficient mice have been reported to develop autoimmunity despite blockade of RT (Achleitner et al., 2017; Stetson et al., 2008a). Of note, RNase H2 has been reported to promote rather than repress retrotransposition in human cell lines (Bartsch et al., 2017; Benitez-Guijarro et al., 2018). In addition, the role of astrocytes in other AGS mutations remains to be addressed.

We address here the impact of two distinct AGS-causing gene defects, *TREX1* and *RNASEH2B*, on iPSC-derived astrocytes and neurons using both isogenic genome-edited and patient-derived cells. Our work identifies DNA damage as a key trigger of neurotoxic inflammatory programs in AGS astrocytes and points toward a previously unappreciated role of AGS genes in R-loop homeostasis. Furthermore, we identify pharmacologically targetable molecular pathways involved, paving the way for the development of strategies to counteract disease pathogenesis.

## Results

### AGS neural stem cells (NSCs)/progenitor cells do not harbor spontaneous signs of disease

**Figure 1.**
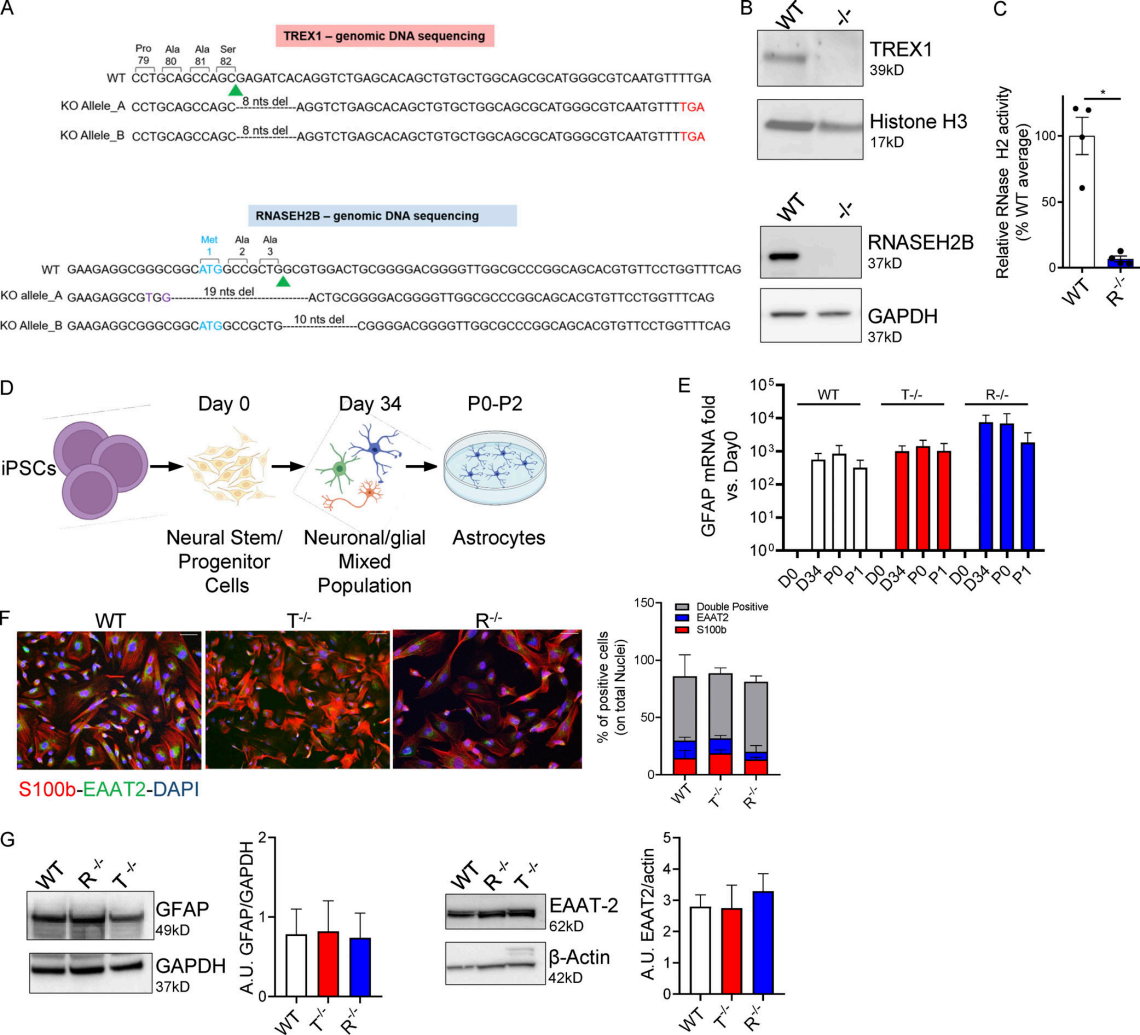
**KO iPSCs efficiently differentiate into proinflammatory astrocytes. (A)** Human genomic DNA sequencing. Green arrowhead, sgRNA cut site; purple, nucleotide substitutions; cyan, start codon; red, premature stop codon generated by nonhomologous end joining–mediated out-of-frame repair. Nucleotides deletions are dashed. Reported are the amino acids encoded by the nucleotides upstream of the sgRNA cut site. **(B)** WB detection of TREX1 and RNASEH2B protein levels in WT and edited clones. (Bottom panel also shown in Fig. S1 A.) **(C)** RNase H2 enzymatic activity was analyzed in WT and KO iPSCs as cleavage of an in vitro synthetized and labeled RNA/DNA molecule measured by fluorescence. (Mean ± SEM; *n* = 4 independent differentiations; one-tailed Mann–Whitney *U* test; *, P < 0.05.) **(D)** Scheme of proinflammatory astrocytes differentiation protocol created with BioRender.com. **(E)** GFAP astrocyte marker was detected by gene expression in full KO clones and WT at passage 1 after the iPSC-derived astrocytes enrichment step and expressed as fold vs. day 0, normalized to the HPRT1 housekeeping gene. (Mean ± SEM; *n* = 5 independent differentiations.) **(F)** Percentages of S100b- and EAAT2-expressing double-positive cells were measured by IF at passage 1 and quantified. One representative image per genotype is shown. Scale bar, 50 µm. (Mean ± SEM; *n* = 4 independent differentiations.) **(G)** Expression and ImageJ quantification of the astrocyte markers EAAT2 and GFAP by WB at passage 1. One representative gel is shown. (Mean ± SEM; *n* = 3 independent differentiations.) Source data are available for this figure: SourceDataF1.

**Figure S1.**
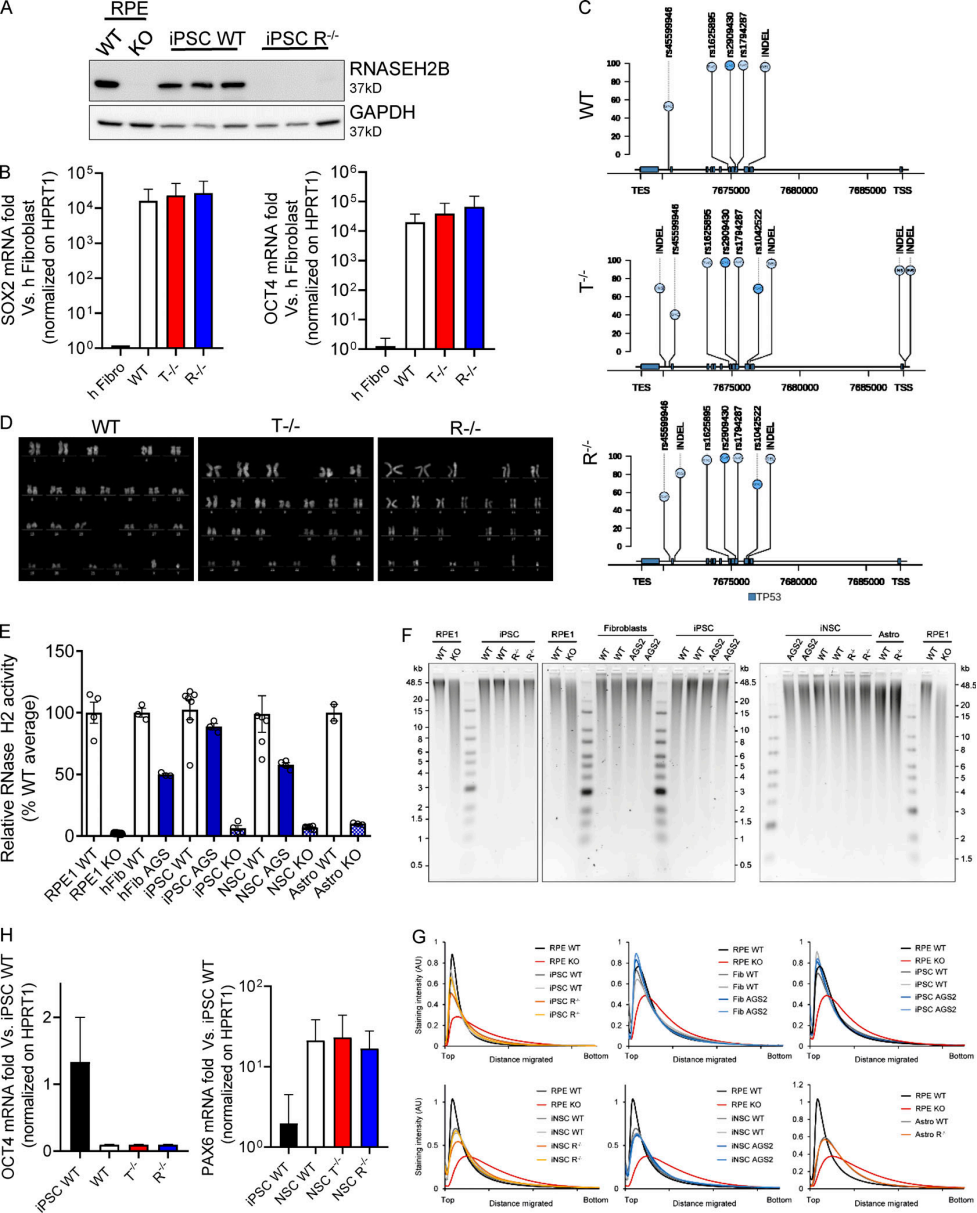
**Characterization of KO iPSCs and iPSC-NSCs. (A)** Loss of RNASEH2B expression in RNASEH2BKO iPSCs was confirmed by WB and comparison to RNASEH2BKO hTERT-RPE1 cells. (*n* = 3 independent experiments; panel also shown in Fig. 1 B.) **(B)** SOX2 and OCT4 pluripotency markers were detected by gene expression in full KO clones and WT compared with the healthy human fibroblasts (h Fibro) previously used to reprogram these iPSCs and normalized on HPRT1 housekeeping gene. (Mean ± SEM; *n* = 3 independent experiments.) **(C)** Lolliplot of TP53 variants in WT, T^−/−^, and R^−/−^ KO clones. Only single nucleotide polymorphisms (SNPs) and insertion–deletion mutations (INDELs) with a frequency higher than the 20% (see y-axis scale) are reported, and variants located in exonic vs. intronic regions are colored in a different way (light blue and bright blue, respectively). TES, transcription end site. **(D)** Karyotype of the WT and KO clones. **(E)** Although cellular RNase H2 activity was substantially reduced in RNASEH2BKO iPSCs, iPSC-NSCs, and astrocytes, as well as in AGS2 (RNASEH2BA177T/A177T) primary fibroblasts, iPSCs and iPSC-NSC KO cells retained low-level nuclease activity against substrate (DNA duplex with a single embedded ribonucleotide), above that seen for independently generated RNase H2–null hTERT-RPE1 cells. Individual data points for *n* = 2–6 independent differentiations. (Mean ± SEM; *n* = 2–6 independent experiments.) **(F)** Representative results for alkaline gel electrophoresis of RNase H2–digested genomic DNA from WT, RNASEH2BKO (R^−/−^), and RNASEH2BA177T/A177T (AGS2) cells (primary fibroblasts, iPSCs, iPSC-NSCs, and astrocytes). hTERT-RPE1 RNase H2–null (RPE1 KO) DNA was included as a positive control for increased fragmentation as a consequence of embedded ribonucleotides. (*n* = 2–3 technical replicates.) **(G)** Densitometry plots for gels in A show no apparent shift in DNA fragment size distribution for mutant RNASEH2B fibroblasts, iPSCs, iPSC-NSCs, or astrocytes, in contrast with independent RNase H2–null RPE1 cells. **(H)** OCT4 and PAX6 were detected by gene expression in differentiated WT and KO clones in comparison with WT iPSCs and normalized on HPRT1 housekeeping gene. (Mean ± SEM; *n* = 3 independent experiments.) Source data are available for this figure: SourceDataFS1.

To model AGS in a physiologically relevant neural context, we generated isogenic iPSCs knocked out for *TREX1* or *RNASEH2B*, starting from a previously well-characterized iPSC clone reprogrammed from healthy donor fibroblasts (Meneghini, 2017). All editing reagents were delivered transiently through electroporation, followed by limiting dilution of single clones, of which we isolated ∼30–40 per condition for molecular screening. All clones were analyzed by mismatch-sensitive molecular assay, Sanger sequencing of individual alleles obtained through TOPO TA cloning assay, and Western blot (WB). One biallelic KO clone per gene was selected for further analysis. A clone obtained from the screen harboring the WT genome was selected as a WT reference for all further experiments (Fig. 1, A and B; and Fig. S1 A). We did not observe any statistically significant differences in the mRNA expression of the pluripotency markers OCT4 and SOX2 between WT *RNASEH2B*^−/−^ (R^−/−^) and *TREX1*^−/−^ (T^−/−^) KO clones (Fig. S1 B).

As the efficiency of precise genome engineering in human iPSCs with a WT p53 gene has been reported to be severely reduced (Ihry et al., 2018), and they can acquire p53 mutations (Merkle et al., 2017), we verified that our KO clones showed normal karyotype and lacked mutations altering the p53 gene sequence (Fig. S1, C and D). Substantial loss of RNase H2 activity in the RNASEH2B KO cells was confirmed using a RNase H2–specific nuclease activity assay (Figs. 1 C and S1 E). As some RNase H2 activity was detected in the KO iPSCs and derived cells, at a level slightly above that seen for RNase H2–null RPE1 cells (Fig S1 E), the R^−/−^ iPSCs may not have complete loss-of-function. Consistent with this possibility, no increased fragmentation was detected by alkaline gel electrophoresis for the R^−/−^ cells used in this work (Fig. S1, F and G). This suggests that they do not have the same level of genome-embedded ribonucleotides that are a general feature of cells without any RNase H2 activity (e.g., RPE1; Fig. S1, F and G). Of note, RNASEH2B-deficient AGS2 patient–derived fibroblasts, iPSCs, and derived cells show >50% of RNaseH2 activity and no increased fragmentation on alkaline gel electrophoresis, remaining well above the levels detected in the cells we generated here that have lost >90% of the normal level of RNase H2 activity (Fig. 1 C and Fig. S1, E–G). Thus, the edited clones will be referred to as RNASEH2B KO or R^−/−^ throughout the article.

**Figure S2.**
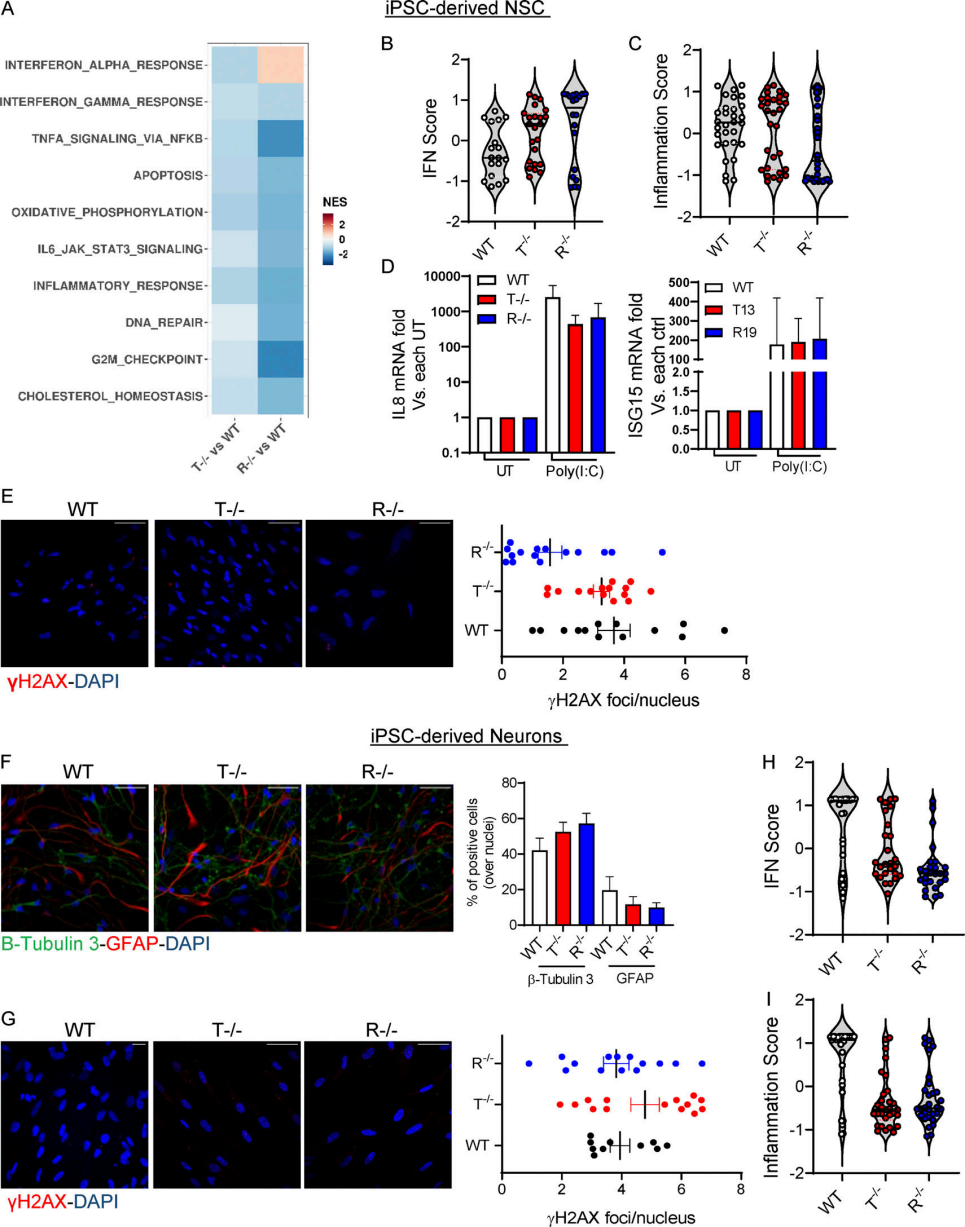
**KO iPSC-NSCs and iPSC-derived neurons do not show strong activation of inflammation or DNA damage responses. (A)** Heatmap visualizing the significantly enriched GSEA terms in iPSC-derived NSCs against the Hallmark gene set (Molecular Signatures Database). GSEA was performed on logFC preranked gene lists obtained from T^−/−^ and R^−/−^ gene expression compared with WT. (NES, normalized enrichment score; adjusted P < 0.05.) **(B and C)** Violin plots showing the distribution of IFN score (B) and inflammation score (C) in T^−/−^ and R^−/−^ KO cells measured from the RNA-seq expression data in NSCs of 32 ISGs and 30 IRGs, respectively, compared with WT. **(D)** IL8 and ISG15 mRNA expression levels in iPSC-derived iNSCs stimulated with poly(I:C) for 6 h. Data are expressed as fold difference vs. untreated condition and normalized to HPRT1 housekeeping gene. (Mean ± SEM; *n* = 3 technical replicates; ctrl, control; UT, untreated.) **(E)** IF of WT and KO iPSC–derived NSCs stained for γH2AX quantified by cell profiler. Each dot corresponds to one field from three slides of four independent differentiations. Scale bar, 50 µm. (Mean ± SEM; *n* = 4 independent experiments.) **(F)** Percentages of cells expressing Tuj1 neuronal marker and GFAP glial marker were measured and quantified upon neuronal differentiation of WT and KO iPSC-NSCs. Three slides from two independent differentiations were analyzed. One representative image per genotype is shown. Scale bar, 50 µm. (Mean ± SEM; *n* = 2 independent experiments.) **(G)** IF of WT and KO iPSC–derived neurons stained for γH2AX quantified by cell profiler. Each dot corresponds to one field from three slides of four independent differentiations. Scale bar, 50 µm. (Mean ± SEM; *n* = 4 independent experiments.) **(H and I)** Violin plots showing the distribution of IFN score (H) and inflammation score (I) in T^−/−^ and R^−/−^ KO cells measured from the RNA-seq expression data in neurons of 32 IFN-RGs and 30 IRGs, respectively, compared with WT.

The KO and WT iPSC clones were then differentiated into induced iPSCs-NSCs (Chambers et al., 2009). The neural induction resulted in down-regulation of the pluripotency gene OCT4 and up-regulation of the neural marker PAX6, observed by quantitative RT-PCR (qRT-PCR; Fig. S1 H). Substantial loss of RNase H2 activity was confirmed in RNASEH2B KO iPSC-NSCs (Fig. S1 E). To characterize the differentiated iPSC-NSCs, we performed genome-wide transcriptomic analysis on KO and WT cells (Fig. S2 A and Table S5). Although type I IFN, proinflammatory, and DNA damage responses have all been associated with AGS gene defects in other cellular models (Crow et al., 2015a; Lim et al., 2015; Nakad and Schumacher, 2016), none of these responses were significantly upregulated in KO iPSC-NSCs in a pathway enrichment analysis (Fig. S2 A). To further dissect activation of type I IFN and inflammatory responses in KO iPSC-NSCs, we calculated specific IFN and inflammation scores using sets of 30 and 32 genes, respectively, as previously described (Kim et al., 2018) and further detailed in Materials and methods. Although there was a trend toward an increased IFN score in TREX1 and RNASEH2B KO iPSC-NSCs (Fig. S2 B), no differences were observed in the Inflammation score between WT and KO cells (Fig. S2 C). Of note, both WT and KO iPSC-NSCs were equally responsive to the TLR3 agonist Poly (I:C) (Fig. S2 D). Although Poly (I:C), as a dsRNA molecule, is not the direct substrate of the AGS-causing genes studied here, it elicited robust responses in KO cells, ruling out that lack of significant innate immune activation in these cells could be due to a broader impairment in innate immune sensing. In addition, no signs of increased DNA damage were observed in KO iPSC-NSCs as detected by the accumulation of nuclear foci for the phosphorylated form of the γH2AX (Fig. S2 E). Taken together, these results suggest that AGS-related phenotypes are not robustly detected in iPSC-NSCs in the context of TREX1 or RNASEH2B deficiencies.

### AGS iPSC–derived astrocytes spontaneously activate type I IFN and proinflammatory responses

**Figure 2.**
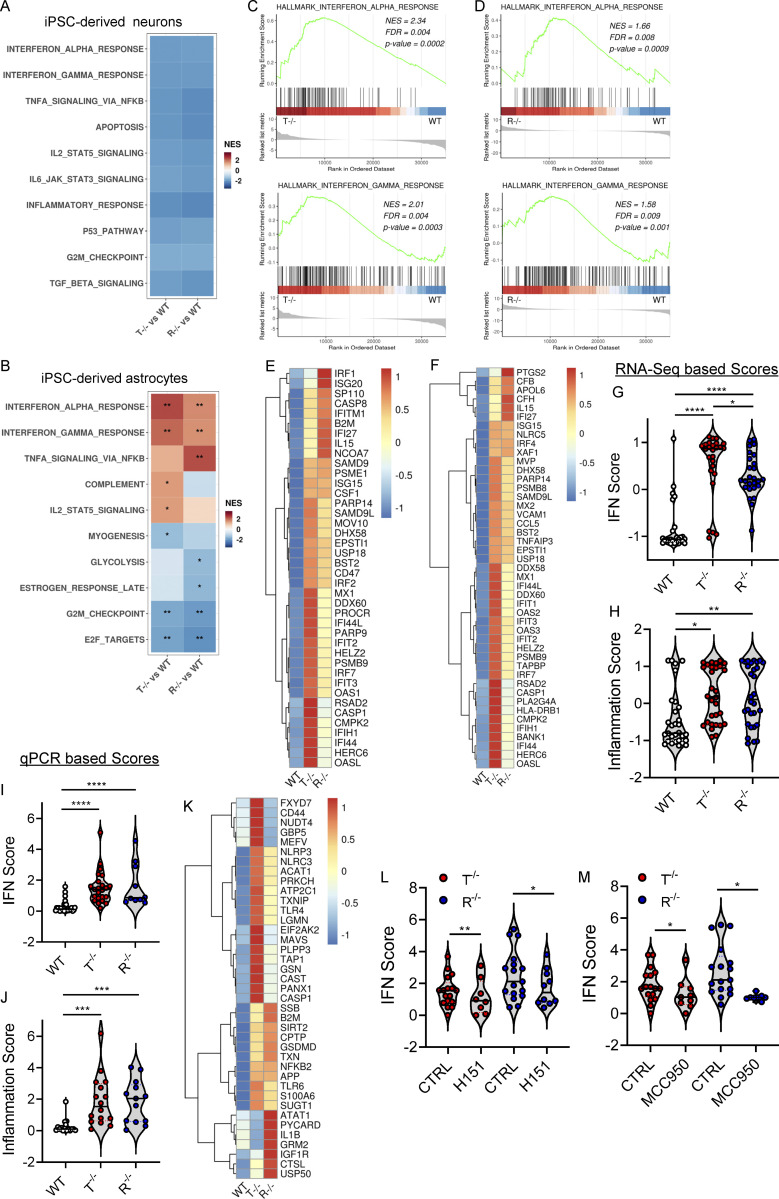
**KO iPSC–derived astrocytes spontaneously activate type I IFN and proinflammatory responses. (A and B)** Heatmap visualizing the significant enriched GSEA terms in iPSC-derived neurons (A) and iPSC-derived astrocytes (B) against the Hallmark gene set (Molecular Signatures Database). GSEA was performed on logFC preranked gene lists obtained from T^−/−^ and R^−/−^ gene expression compared with WT. (NES, normalized enrichment score; *, adjusted P < 0.05; **, adjusted P < 0.01 for upregulated pathways.) **(C and D)** Enrichment plots showing the GSEA results of the hallmark IFNα response gene set (C) and hallmark IFNγ response gene set (D) resulting from the comparison of genes preranked based on logFC values in T^−/−^ KO cells vs. WT (upper plot) and R^−/−^ KO cells vs. WT (lower plot) in astrocytes. The top part of each plot shows the enrichment score representing the running-sum statistic calculated by walking down the ranked list of genes. The middle part shows the position of a member of the gene set in the ranked list of genes. The bottom part depicts the ranking metric that measures a gene’s correlation with the biological function. **(E and F)** Heatmaps depicting the expression levels of genes belonging to IFNα (E) and IFNγ (F) gene sets upregulated in T^−/−^ cells and R^−/−^ cells. Gene expression, in rows, was row-scaled (z-scores) for visualization. The color scale differentiates values as high (red), medium (yellow), and low (blue) expression. **(G and H)** Violin plots showing the distribution of IFN score (G) and the inflammation score (H) in T^−/−^ and R^−/−^ KO cells in astrocytes measured from the RNA-seq expression data of 32 IFN-RGs and 30 IRGs, respectively, compared with WT. (Wilcoxon rank-sum test; *, P < 0.05; **, P < 0.01; ****, P < 0.0001.) **(I and J)** Violin plots showing the IFN score (I) and inflammation score (J) measured in T^−/−^ and R^−/−^ KO cells from qRT-PCR quantification of the median FC of six ISGs and six IRGs, respectively. (*n* = 5 independent experiments; Wilcoxon rank-sum test; ***, P < 0.001; ****, P < 0.0001.) **(K)** Heatmap showing the expression level of genes belonging to the NLRP3 inflammasome. Gene expression, in rows, was row-scaled (z-scores) for visualization. **(L and M)** Violin plots showing the distribution of IFN scores calculated from the median FC of six ISGs in T^−/−^ and R^−/−^ KO cells treated with the STING inhibitor H151 (L) or NLRP3 inhibitor MCC950 (M). (*n* = 3 independent experiments; Wilcoxon rank-sum test; *, P < 0.05; **, P < 0.01.)

To study the impact of AGS gene deletions in more committed cells of the CNS, the iPSC-NSCs were differentiated in vitro into inflammation-responsive astrocytes as previously reported (Santos et al., 2017) and toward neurons according to a commercial differentiation protocol. The astrocyte differentiation protocol was optimized for monolayer culture (Fig. 1 D) and applied to the WT and KO iPSC clones. Differentiation efficiency was assessed in terms of glial fibrillary acidic protein (GFAP) mRNA levels and S100b and EAAT2 expression by immunofluorescence (IF), as well as WB for iPSC-derived astrocytes (Fig. 1, E–G); β-Tubulin III was measured by IF in iPSC-derived neurons (Fig. S2 F). All three clones were equally able to differentiate into iPSC-derived astrocytes and neurons (Fig. 1, E and F; and Fig. S2 F). To characterize the transcriptional changes in differentiated KO iPSC–derived astrocytes and neurons, we performed genome-wide transcriptomic analysis (Fig. 2, A and B; and Table S5). Similar to KO iPSC-NSCs (Fig. S2 A), no significant upregulation for pathways involving type I IFN, proinflammatory, or DNA damage responses was observed for KO iPSC–derived neurons (Fig. 2 A). In agreement, no increase in nuclear γH2AX foci was detected (Fig. S2 G), and the IFN and inflammatory scores were lower than those observed in WT cells (Fig. S2, H and I).

In contrast, differences in type I IFN and proinflammatory signatures started to emerge between WT and KO iPSC–derived astrocytes (Fig. 2 B). Both TREX1- and RNASEH2B-deficient cells showed a significant activation of IFNα- and IFNγ-mediated responses in the absence of any exogenous triggers (Fig. 2 B). This was confirmed by specific gene set enrichment analysis (GSEA; Fig. 2, C–F) and IFN scores calculated from the transcriptomic data (Fig. 2 G) and further validated by qRT-PCR of a selected set of genes (Lambers et al., 2019; Rice et al., 2013; Rice et al., 2018; Tungler et al., 2016; Fig. 2 I). In line with the observed enrichment for proinflammatory pathways, such as TNFα via NF-κB, and complement-mediated responses in the KO iPSC–derived astrocytes (Fig. 2 B), these cells showed significant upregulation of several genes associated with NLRP3 inflammasome activation (Ising et al., 2019; Ju et al., 2021; Li and Liu, 2015; Zhou et al., 2016; Fig. 2 K) and an increased inflammation score (Fig. 2, H–J). In addition, exposure of KO iPSC–derived astrocytes to the stimulator of IFN genes (STING) inhibitor H151 (Haag et al., 2018) or the NLRP3-specific inhibitor MCC950 (Coll et al., 2015) significantly reduced IFN scores in both TREX1 and RNASEH2B KO cells (Fig. 2, L and M), indicating that both pathways contribute to this signature in AGS astrocytes.

Taken together, these data establish that type I IFN and several proinflammatory signaling cascades are spontaneously activated in both *TREX1* and *RNASEH2B* KO iPSC–derived astrocytes and that both STING and NLRP3-related pathways contribute to the increased IFN scores in these cells.

### Single-cell analysis of patient-derived mixed neural populations reveals transcriptional alterations across cell types

**Figure 3.**
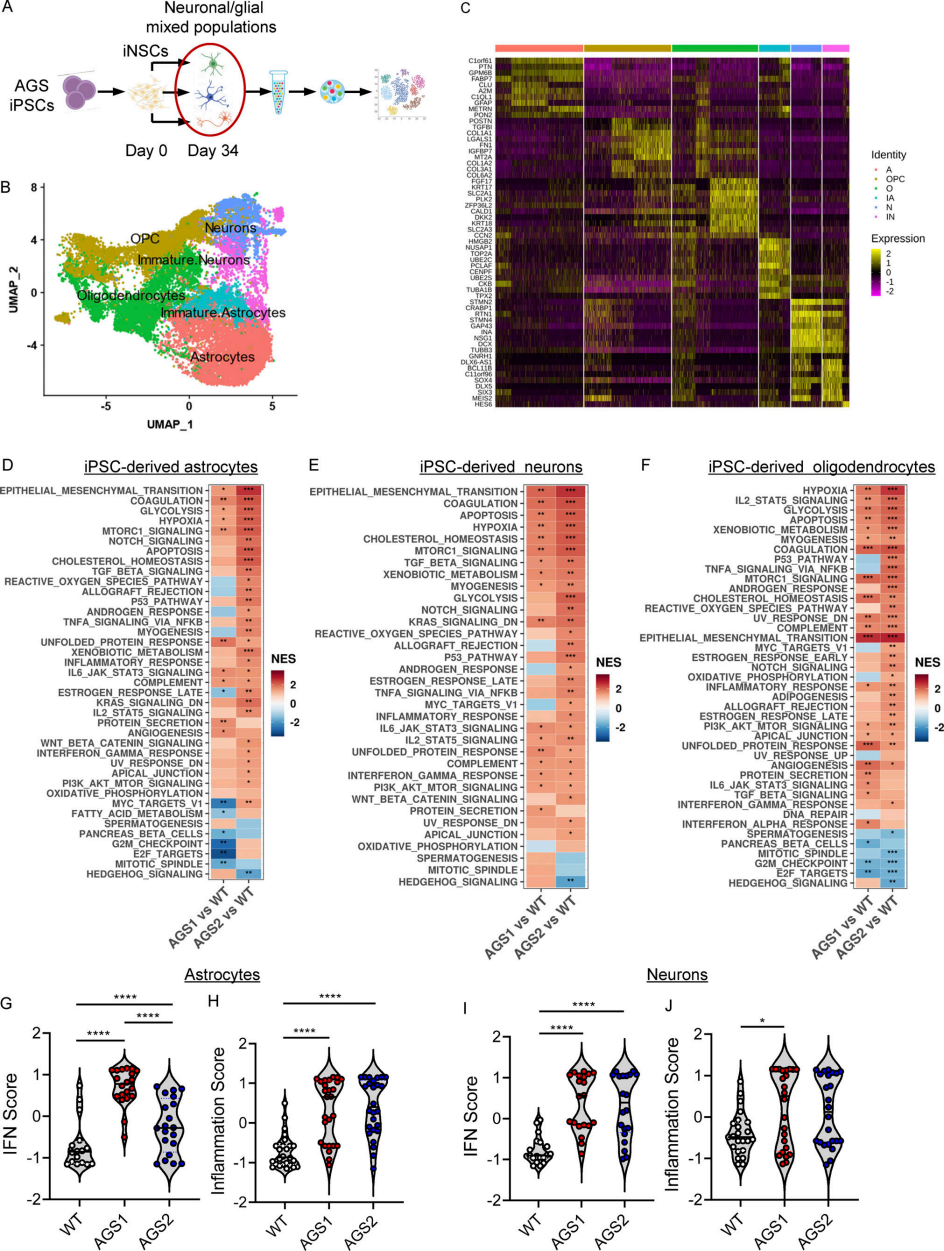
**Single-cell transcriptomics reveals AGS phenotypes in patient-derived neuronal/glial mixed populations. (A)** scRNA-seq experimental scheme (created with BioRender.com) performed with iPSC-derived cells from patients harboring mutations in TREX1 (AGS1) or RNASEH2B (AGS2). **(B)** UMAP plot of single-cell data with Seurat. Cells are clustered in two dimensions using the UMAP dimensionality reduction technique and annotated by cell type. **(C)** Hierarchically clustered average gene expression heatmap for genes overexpressed across the different cell types grouped according to the Seurat classification (A, astrocytes; IA, immature astrocytes; IN, immature neurons; N, neurons; O, oligodendrocytes; OPC, oligodendrocyte precursor cells). Yellow, high expression; purple, low expression. Scaled-in normalized gene expression data are shown. **(D–F)** Heatmap visualizing the enriched GSEA terms in astrocytes (D), neurons (E), and oligodendrocytes (F) against the Hallmark gene set (Molecular Signatures Database). GSEA was performed on logFC preranked gene lists obtained from AGS1 and AGS2 gene expression compared with WT within each cell type. (NES, normalized enrichement score; *, adjusted P < 0.05; **, adjusted P < 0.01; ***, adjusted P < 0.001.) **(G–J)** Violin plots showing the distribution of IFN scores and inflammation scores in astrocytes (G and H) and neurons (I and J) calculated from the expression of 32 IFN-RGs and 30 IRGs, respectively, in AGS1 and AGS2 patients compared with WT. (Wilcoxon rank-sum test; *, P < 0.05; ****, P < 0.0001.)

**Figure S3.**
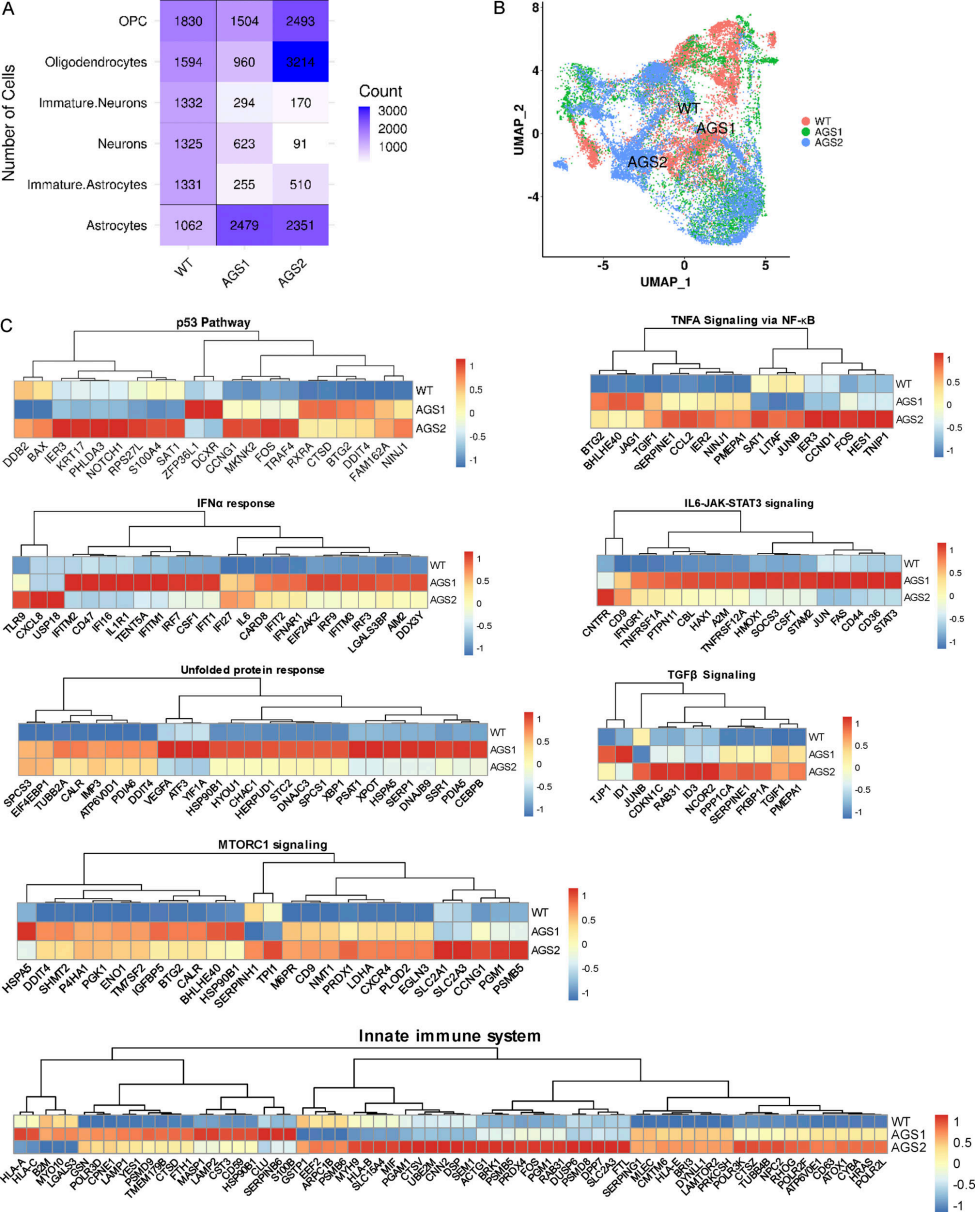
**Spontaneous activation of multiple inflammatory pathways identified by scRNA-seq in AGS patient–derived astrocytes. (A)** Number of cells quantified from single-cell data by CellRanger in the different cell type clusters across the studied samples. **(B)** UMAP plot showing the clustering of cells across samples with Seurat. Cells are clustered in two dimensions using the UMAP dimensionality reduction technique and annotated by sample type. **(C)** Heatmaps depicting the expression of genes from the GSEA terms identified from the different comparisons between AGS1 and AGS2 iPSC-derived astrocytes compared with WT. Gene expression, in rows, was row-scaled (z-scores) for visualization. The color scale differentiates values as high (red), medium (yellow), and low (blue) expression.

To further investigate activation of the above pathways in the context of patient-derived cells, we differentiated iPSC-NSCs derived from patients harboring mutations in *TREX1* (AGS1: compound heterozygous for p.R97H and p.S88fs*22; Ferraro et al., 2019a) or *RNASEH2B* (AGS2: homozygous for p.A177T; Ferraro et al., 2019b) into mixed neuronal/glial cell populations (Frati et al., 2018; Santos et al., 2017). These TREX1 mutations have been reported to associate with microcephaly, chilblains-like lesions, severe tetraparesis, cerebral calcifications, leukodystrophy, and raised cerebrospinal fluid IFNα, all typical clinical features of AGS (Olivieri et al., 2013). The A177T mutation is known to cause reduced RNase H2 complex stability (Figiel et al., 2011; Reijns et al., 2011), and AGS2 cells have a modest but significant decrease in cellular RNase H2 activity (Fig S1 E), consistent with a previous report (Mackenzie et al., 2016). To simultaneously interrogate transcriptomes in the different cell populations, we performed single-cell RNA sequencing (scRNA-seq), comparing cells from AGS patients to healthy donor control cells derived from iPSCs generated through similar technology (Ferraro et al., 2019a; Ferraro et al., 2019b; Fig. 3 A and Table S6). We were able to identify clusters of cells harboring gene signatures characteristic of immature and more committed neurons, astrocytes, and oligodendrocytes in all samples (Keil et al., 2018; Tanaka et al., 2020; Fig. 3, B and C; and Fig. S3, A and B).

The transcriptional changes observed within the AGS patient iPSC–derived astrocytes in this context confirmed alterations in several proinflammatory pathways and revealed significant modulation of many others involved in DNA damage and metabolic responses (Figs. 3 D and S3 C). Although no defects in neuronal differentiation were observed in the KO setting using a neuron-specific differentiation protocol (Fig. S2 F), single-cell analysis of patient iPSC–derived neurons from a mixed population revealed dysregulation of apoptotic and inflammatory pathways both in the neuronal population and in oligodendrocytes (Fig. 3, E and F). This suggests that the presence of the astroglial component during differentiation significantly contributes to activating the transcriptional programs also in these CNS cell subsets. In addition, several metabolic pathways were significantly altered in all AGS patient–derived cell populations (Figs. 3 D and S3 C). Among these, glycolytic, hypoxic, and mTORC1 signaling pathways were upregulated across AGS genotypes (Fig. 3, D–F; and Fig. S3 C). To confirm activation of type I IFN and inflammatory responses, the same IFN and inflammation scores calculated from the transcriptomic analysis of AGS KO iPSC–derived cells (Fig. 2, G–J) were applied to the patient-derived TREX1- and RNASEH2B-deficient astrocytes and neurons in the mixed culture setting. Both scores were significantly increased in patient-derived astrocytes and neurons (Fig. 3, G–I and L), although slightly lower type I IFN scores were observed for RNASEH2B-deficient astrocytes in line with the IFN scores retrieved for the KO astrocytes (Fig. 2 G).

Taken together, these results reveal spontaneous activation of proinflammatory and DNA damage responses in AGS patient–derived neural populations, highlighting the prominent impact of AGS gene defects on cells of the human CNS and uncovering significant alterations in several metabolic pathways that may play a yet unexplored role in AGS pathogenesis.

### Increased DNA damage contributes to inflammation in AGS iPSC–derived astrocytes

**Figure S4.**
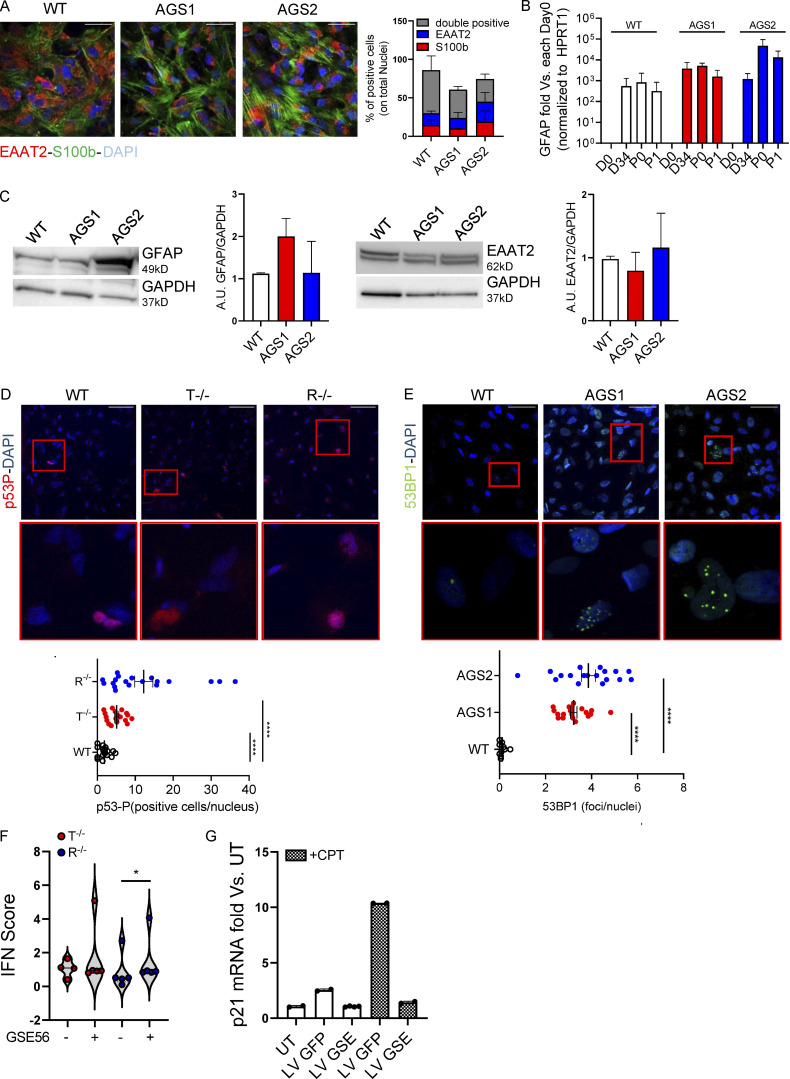
**AGS patient–derived and KO iPSCs efficiently differentiate into proinflammatory astrocytes and spontaneously activate DNA damage responses.** iPSC-derived proinflammatory astrocytes from healthy control (WT) or patients harboring mutations in TREX1 (AGS1) or RNASEH2B (AGS2) were analyzed. **(A)** Percentages of S100b- and EAAT2-expressing and double-positive cells were measured by IF and quantified. One representative image per genotype is shown. Five fields from three slides of two independent differentiation experiments were acquired. Scale bar, 50 µm. (Mean ± SEM; *n* = 2 independent experiments.) **(B)** GFAP astrocyte marker was detected by gene expression in AGS patient–derived clones and WT at passage 1 after the iPSC-derived astrocyte enrichment step and expressed as fold difference vs. day 0, normalized on HPRT1 housekeeping gene. (Mean ± SEM; *n* = 3 independent experiments.) **(C)** Expression and ImageJ quantification of the astrocyte markers EAAT2 and GFAP by WB. One representative gel is shown. (Mean ± SEM; *n* = 3 independent experiments.) **(D)** IF of WT and KO iPSC–derived proinflammatory astrocytes stained for phosphorylated p53, quantified by measuring area intensity normalized on number of nuclei. Each dot corresponds to one field from three slides of four independent differentiations. Scale bar, 50 µm. (Mean ± SEM; *n* = 4 independent experiments; one-tailed Mann–Whitney *U* test; ****, P < 0.0001.) **(E)** IF of AGS patient–derived iPSC-derived proinflammatory astrocytes stained for 53BP1, quantified by cell profiler. Each dot corresponds to one field from three slides of three independent differentiations. (Mean ± SEM; *n* = 3 independent experiments; one-tailed Mann–Whitney *U* test; ****, P < 0.0001.) Scale bar, 50 µm. **(F)** Violin plot showing the distribution of IFN scores calculated from the median FC of six ISGs in T^−/−^ and R^−/−^ KO cells transduced with a lentiviral vector expressing GSE56. (*n* = 2 independent experiments; Wilcoxon rank-sum test; *, P < 0.05.) **(G)** p21 mRNA levels were detected by gene expression in astrocytes upon transduction with lentiviral vector expressing GFP (LV GFP) or GSE (LV GSE56) in the presence or not of Camptothecin (CPT), a topoisomerase inhibitor. Data are expressed as fold vs. untransduced cells (UT) and normalized to HPRT1 housekeeping gene. (Mean ± SD; *n* = 2 technical replicates.) Source data are available for this figure: SourceDataFS4.

**Figure 4.**
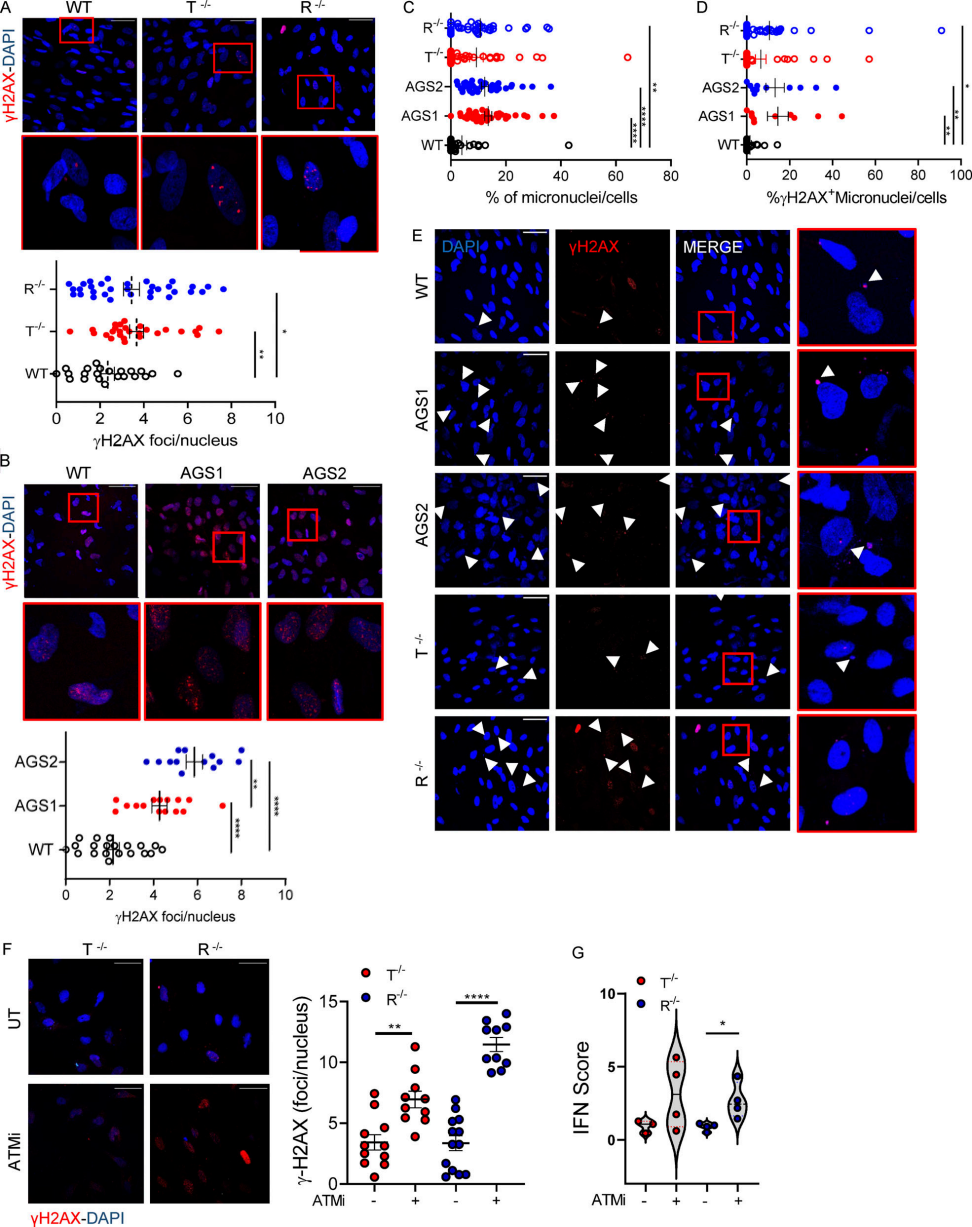
**AGS KO and patient-derived astrocytes show signs of increased DNA damage that contributes to inflammation.** iPSC-derived proinflammatory astrocytes from healthy control (WT), TREX1 KO, RNASEH2B KO, or patients harboring mutations in TREX1 (AGS1) or RNASEH2B (AGS2) were analyzed. **(A)** IF of WT and KO proinflammatory astrocytes stained for γH2AX quantified by cell profiler. Each dot corresponds to one field from three slides of four independent differentiations. Scale bar, 50 µm. (Mean ± SEM; *n* = 4 independent experiments; one-tailed Mann–Whitney *U* test; *, P < 0.05; **, P < 0.01.) **(B)** IF of WT or patient-derived proinflammatory astrocytes stained for γH2AX quantified by cell profiler. Each dot corresponds to one field from three slides of three independent differentiations. Scale bar, 50 µm. (Mean ± SEM; *n* = 3 independent experiments; one-tailed Mann–Whitney *U* test; **, P < 0.01; ****, P < 0.0001.) **(C)** Micronuclei counts in WT, KO, and AGS patient iPSC–derived astrocytes. Each dot corresponds to one field from three slides of three (AGS patients) and five (WT and KO) independent iPSC-derived astrocyte differentiation experiments. (Mean ± SEM; *n* = 3–5 independent experiments; Kruskal–Wallis test; **, P < 0.01; ****, P < 0.0001.) **(D)** γH2AX-positive micronuclei counts in WT, KO, and AGS patient–derived astrocytes. Each dot corresponds to one field from three slides of three (AGS patients) and five (WT and KO) independent astrocyte differentiation experiments. (Mean ± SEM; *n* = 3–5 independent experiments; Kruskal–Wallis test; *, P < 0.05; **, P < 0.01.) **(E)** Representative images of micronuclei and γH2AX-positive micronuclei (white arrowheads) for each sample. Scale bar, 50 µm. **(F)** IF of WT and KO proinflammatory astrocytes treated or not (UT) with ATM inhibitor (ATMi) and stained for γH2AX quantified by cell profiler. Each dot corresponds to one field from three slides of three independent differentiations. Scale bar, 50 µm. (Mean ± SEM; *n* = 3 independent experiments; one-tailed Mann–Whitney *U* test; **, P < 0.01; ****, P < 0.0001.) **(G)** Violin plot showing the distribution of IFN scores calculated from the median FC of six ISGs in T^−/−^ and R^−/−^ KO cells treated with ATM inhibitor (ATMi). (*n* = 3 independent experiments; Wilcoxon rank-sum test; *, P < 0.05.)

To dissect the functional consequences of the transcriptional alterations in astrocytes, we differentiated KO and patient-derived iPSCs into proinflammatory astrocytes as previously described for our KO model (Fig. 1 D). For KO astrocytes, no genotype-related differentiation defects were observed in terms of GFAP, EAAT2, or S100b expression assessed by gene expression, WB, and IF for patient-derived cells (Fig. S4, A–C). We evaluated the activation of DNA damage responses in WT and AGS iPSC–derived astrocytes by measuring phosphorylated histone H2AX (γH2AX), p53 (p53-P), and 53BP1 accumulation by IF. KO iPSC–derived astrocytes showed a significant increase in both γH2AX foci (Fig. 4 A) and p53-P levels (Fig. S4 D). In agreement with the KO model and scRNA-seq analysis of patient cells, AGS patient–derived astrocytes also spontaneously activated DNA damage responses, as measured by γH2AX and 53BP1 accumulation (Figs. 4 B and S4 E). In addition, significantly more micronuclei (total and γH2AX positive) were detected in TREX1- and RNASEH2B-deficient iPSC-NSC astrocytes (Fig. 4, C–E), suggesting their potential contribution to inflammatory responses in AGS. Indeed, micronuclei are emerging as an important source of cytoplasmic DNA, causing activation of the innate immune sensor cyclic GMP-AMP synthase (cGAS; Harding et al., 2017; Mackenzie et al., 2017), ultimately resulting in proinflammatory responses as well as activation of type I IFN responses in multiple contexts (Kwon et al., 2020).

The ataxia-telangiectasia mutated protein kinase (ATM) is critical for genome stability during neural development (McKinnon, 2012; McKinnon, 2017) and was recently shown to be involved in controlling neuropathology associated with loss of RNase H2 in mice (Aditi et al., 2021). Inhibition of ATM resulted in a significant increase in the numbers of γH2AX foci in both TREX1 and RNASEH2B KO iPSC–derived astrocytes (Fig. 4 F), suggesting that ATM is critical to limit DNA damage not only in RNASEH2B- but also TREX1-deficient human iPSC-derived astrocytes. ATM inhibition and associated increase in DNA damage signaling also led to further elevated IFN scores in AGS KO iPSC–derived astrocytes (Fig. 4 G), consistent with a role of DNA damage in the proinflammatory activation of AGS astrocytes.

**Figure 5.**
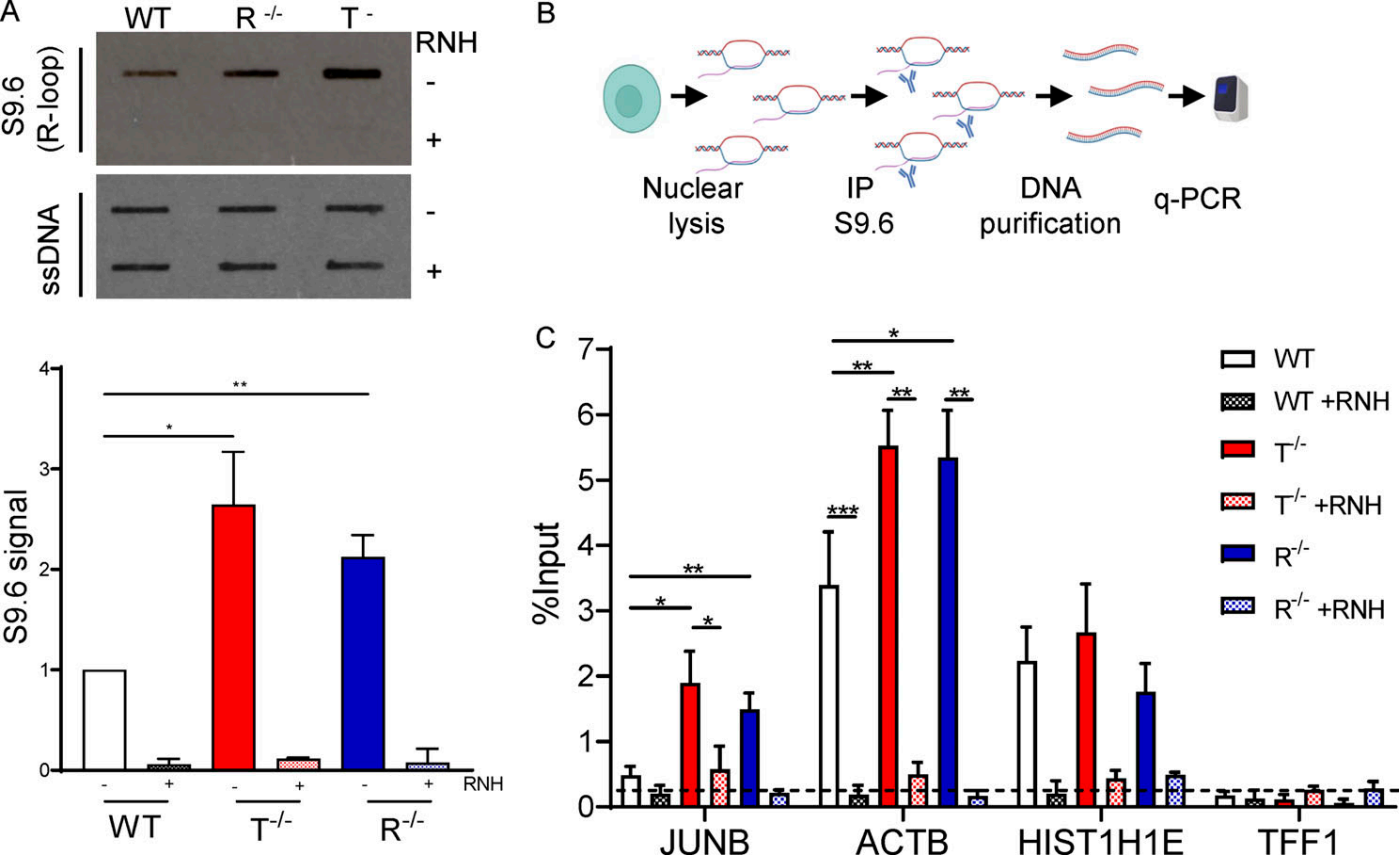
**AGS KO astrocytes accumulate R-loops. (A)** Slot blot analysis of genomic DNA extracted from WT, R^−/−^, and T^−/−^ cells treated with the recombinant RNase H (RNH) enzyme for 2.5 h at 37°C and probed with S9.6 (top) and ssDNA (bottom) antibodies. Slot blot probed with the antibody recognizing ssDNA was used as a loading control. (Mean ± SEM; *n* = 3 independent experiments; two-tailed unpaired *t* test; *, P < 0.05; **, P < 0.01.) **(B)** Scheme of DRIP-qPCR experiment. **(C)** DRIP-qPCR ± RNH treatment for the indicated genes in WT, R ^−/−^, and T^−/−^ cells. Dashed line represents background signal. Data are expressed as percentage of input. (Mean ± SEM; *n* > 3 independent experiments; two-tailed unpaired *t* test; *, P < 0.05; **, P < 0.01; ***, P < 0.001.) Source data are available for this figure: SourceDataF5.

R-loop–regulating factors are associated with autoimmune and neurological disorders, including AGS, suggesting that R-loop imbalance could contribute to disease pathology (Lim et al., 2015; Richard and Manley, 2017). To understand if R-loops are implicated in AGS phenotypes in iPSC-NSC astrocytes, we performed slot blot analyses in WT, *RNASEH2B*^*−/−*^, and *TREX*^*−/−*^ cells (Fig. 5 A). Genomic DNA was extracted from these cells and probed with the S9.6 antibody, which specifically recognizes RNA/DNA hybrids (Boguslawski et al., 1986; Phillips et al., 2013). Remarkably, we observed a significant increase in the global level of nuclear RNA/DNA hybrids in both KO iPSC-NSC astrocytes, reaching a ∼2.0-fold increase in *RNASEH2B*^*−/−*^ and ∼2.5-fold increase in *TREX*^*−/−*^ cells compared with WT cells (Fig. 5 A). Importantly, the S9.6 signal was abolished by RNase H treatment, indicating that it is specific to RNA/DNA hybrids. To investigate whether these R-loops accumulate at the level of specific genes, we performed RNA/DNA hybrid immunoprecipitation (DRIP) with the S9.6 antibody on nuclear DNA extracted from the KO astrocytes, followed by qRT-PCR of selected genomic regions (Cristini et al., 2018). DRIP-qPCR analysis revealed an increased R-loop signal over intronless JUNB and intron-containing ACTB genes in R*NASEH2B*^*−/−*^ and *TREX*^*−/−*^ iPSC-NSC astrocytes (Fig. 5, B and C). The DRIP signal was RNase H sensitive, confirming specificity, and background levels of R-loops were detected over the nontranscribed TFF1 gene. Together, these results suggest that defects in both RNASEH2B and TREX1 associate with significant DNA damage and deregulated R-loop homeostasis in iPSC-NSC–derived astrocytes that contribute to the observed inflammatory responses.

### AGS iPSC–derived astrocytes mediate neurotoxicity that can be pharmacologically rescued

**Figure 6.**
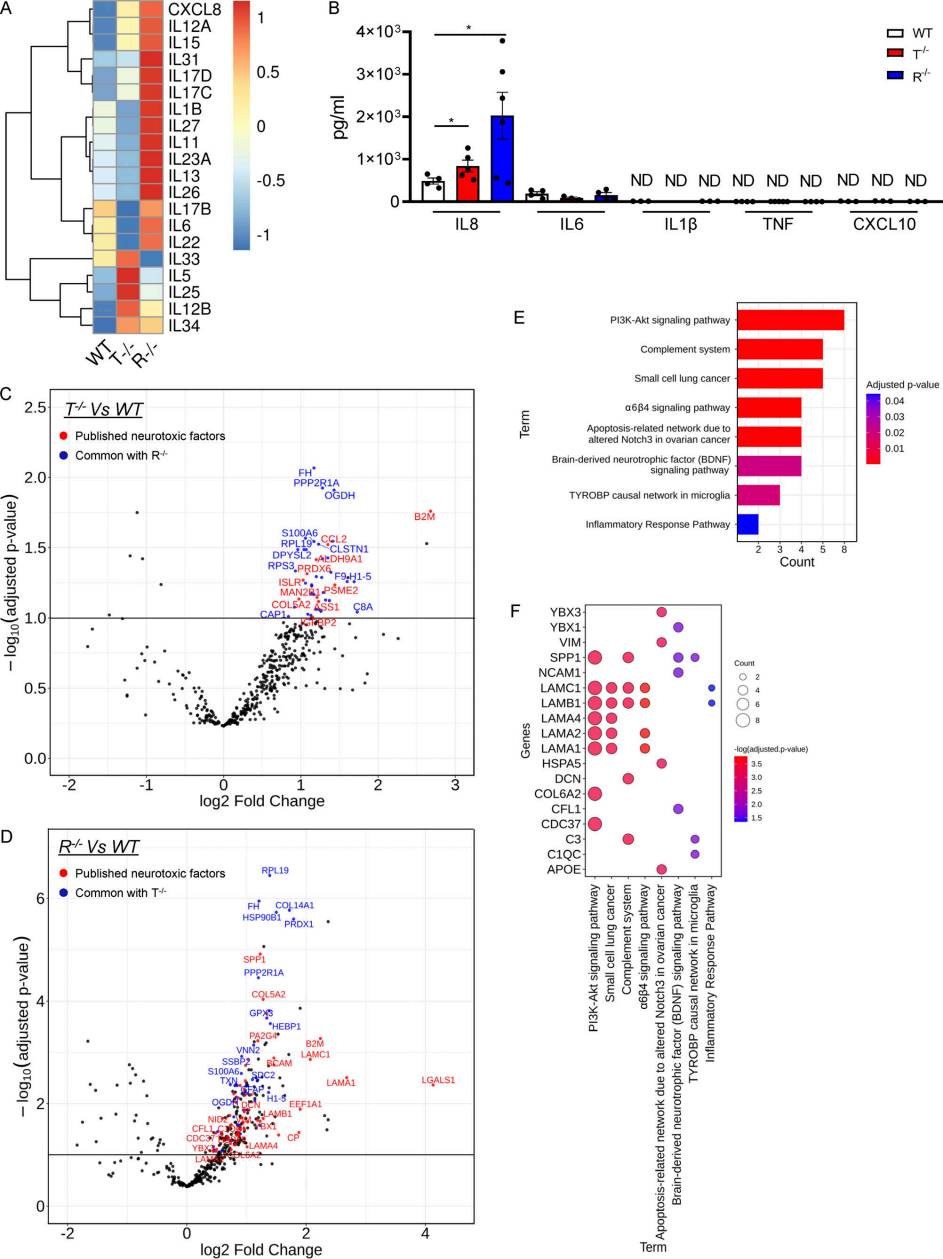
**KO iPSC–derived astrocytes secrete neuroinflammatory mediators. (A)** Heatmap representation showing gene expression analysis of interleukin genes in T^−/−^ and R^−/−^ KO cells compared with WT. **(B)** ELISA results for inflammatory cytokines (IL8, IL6, IL1β, TNFα, and CXCL10) in the supernatant of WT and KO astrocytes at steady state. (Mean ± SEM; *n* = 3 independent experiments; one-tailed Mann–Whitney *U* test; *, P < 0.05.) **(C and D)** Volcano plots highlighting the differentially secreted proteins in R^−/−^ (C) and T^−/−^ (D) compared with WT samples. Log2(FC) and adjusted P values are reported for each protein. Upregulated common secreted proteins between the two comparisons are highlighted in blue. Proteins common with published neurotoxic factors are highlighted in red. **(E)** Barplot highlighting the significant enriched functional pathways resulting from the WikiPathways database of secreted proteins common with published neurotoxic factor. **(F)** Bubble plot showing the differentially secreted neurotoxic factors involved in the identified enriched pathways.

In agreement with the activation of proinflammatory programs, several interleukins were upregulated at the transcriptional level in TREX1 and RNASEH2B KO iPSC–derived astrocytes (Fig. 6 A). However, Luminex-based ELISA assays did not detect many of the cytokines tested, with the exception of the proinflammatory cytokine IL-8, which was present at significantly increased levels for TREX1 and RNASEH2B KO astrocytes (Fig. 6 B), in line with the observed upregulation of TNFα via NF-κB–mediated responses (Fig. 2 B). To explore the possibility that other damage-associated molecular patterns or secreted proteins could associate with the inflammatory status of these cells, we performed mass spectrometry (MS)–based high-throughput secretome analysis of supernatants from WT and KO iPSC–derived astrocytes. A total of 390 secreted proteins among all cell types were identified, with 54 and 270 proteins present at significantly higher levels for TREX1 and RNASEH2B KO cells, respectively (Fig. 6, C and D; and Table S7). Among these, several proteins have been previously described as part of secretory profiles in neuroinflammatory conditions, including spinal cord injury (Didangelos et al., 2016) or neurotoxic and inflammatory astrocytes (Guttenplan et al., 2021; Rooney et al., 2021; Fig. 6, C and D, highlighted in red). Interestingly, the proteins identified in common between the secretomes of the KO iPSC–derived astrocytes and these other neuroinflammatory contexts are significantly enriched for pathways such as the Complement system, inflammatory responses, PI3K-Akt signaling, and pathways involved in neurological injuries or pathologies (Fig. 6, E and F), in agreement with the enriched pathways in our transcriptomic analysis (Fig. 2).

**Figure 7.**
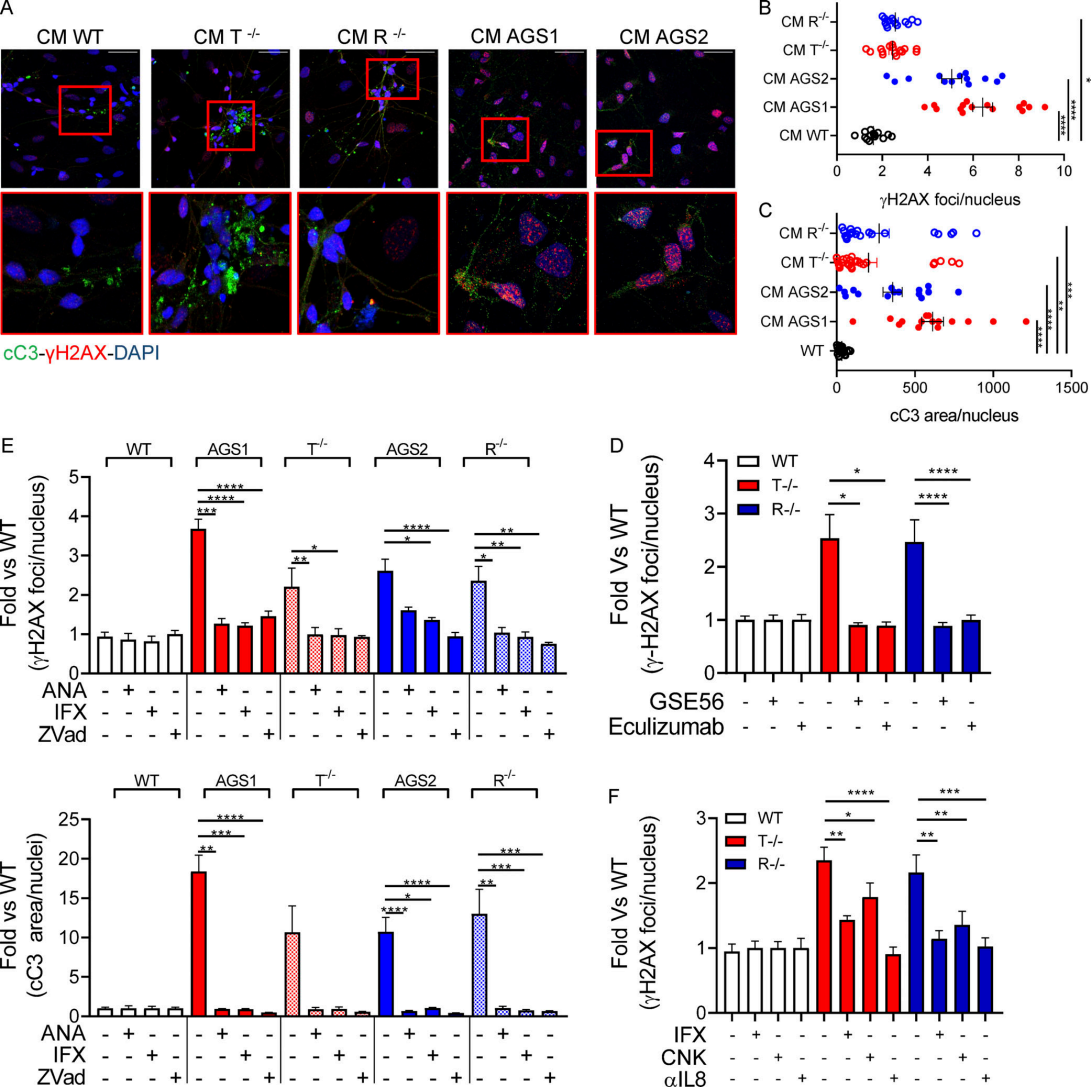
**KO and AGS patient iPSC–derived astrocytes mediate neurotoxicity. (A–C)** WT neurons expression of cleaved Caspase-3 (cC3) and phosphorylated γH2AX after 48-h exposure to KO iPSC–derived astrocyte conditioned medium, measured by IF staining. Representative pictures and quantification. Each dot corresponds to one field from three slides of three independent differentiations. Scale bar, 50 µm. (Mean ± SEM; *n* = 3 independent experiments; Kruskal–Wallis test; *, P < 0.05; **, P < 0.01; ***, P < 0.001; ****, P < 0.0001.) CM, conditioned medium. **(D)** Reduced γH2AX upon exposure of WT neurons to conditioned medium collected from WT and TREX1- or RNASEH2B-deficient astrocytes and then supplemented with Complement C5 inhibitor eculizumab or conditioned medium collected from WT and TREX1- or RNASEH2B-deficient astrocytes overexpressing dominant-negative p53-inhibiting peptide GSE56. (Mean ± SEM; *n* = 3 independent experiments; one-tailed Mann–Whitney *U* test; *, P < 0.05; ****, P < 0.0001.) **(E)** Reduced γH2AX and cleaved Caspase-3 immunopositive area upon exposure of WT neurons to conditioned medium collected from astrocytes treated with anakinra (ANA, IL-1β inhibitor), infliximab (IFX, anti-TNFα antibody), or Z-Vad (pan-caspase inhibitor) for 24 h. γH2AX and cleaved Caspase-3 levels expressed as over WT condition. (Mean ± SEM; *n* = 3 independent experiments; Kruskal–Wallis test; *, P < 0.05; **, P < 0.01; ***, P < 0.001; ****, P < 0.0001.) **(F)** Reduced γH2AX immunopositive area upon exposure of WT neurons to conditioned medium collected from astrocytes supplemented with anti-TNFα (infliximab, IFX), anti–IL-1β (canakinumab, CNK), or anti-IL8 for 48 h. γH2AX expressed as fold over WT condition. (Mean ± SEM; *n* = 3 independent experiments; one-tailed Mann–Whitney *U* test; *, P < 0.05; **, P < 0.01; ***, P < 0.001; ****, P < 0.0001.)

To test whether the inflammatory programs activated in AGS iPSC–derived astrocytes directly contribute to neuroinflammation, we exposed WT iPSC-derived neurons to conditioned medium from AGS KO and patient iPSC–derived astrocytes and assessed signs of toxicity. Importantly, neurons exposed to conditioned medium from TREX1 or RNASEH2B KO iPSC–derived astrocytes showed increased H2AX histone phosphorylation, with even more pronounced toxicity observed in neurons exposed to conditioned medium from AGS patient iPSC–derived astrocytes (Fig. 7, A and B). Similarly, increased cleaved Caspase-3 was detected in neurons exposed to conditioned medium from KO or patient-derived astrocytes (Fig. 7, A and C), indicating elevated levels of apoptosis.

**Figure S5.**
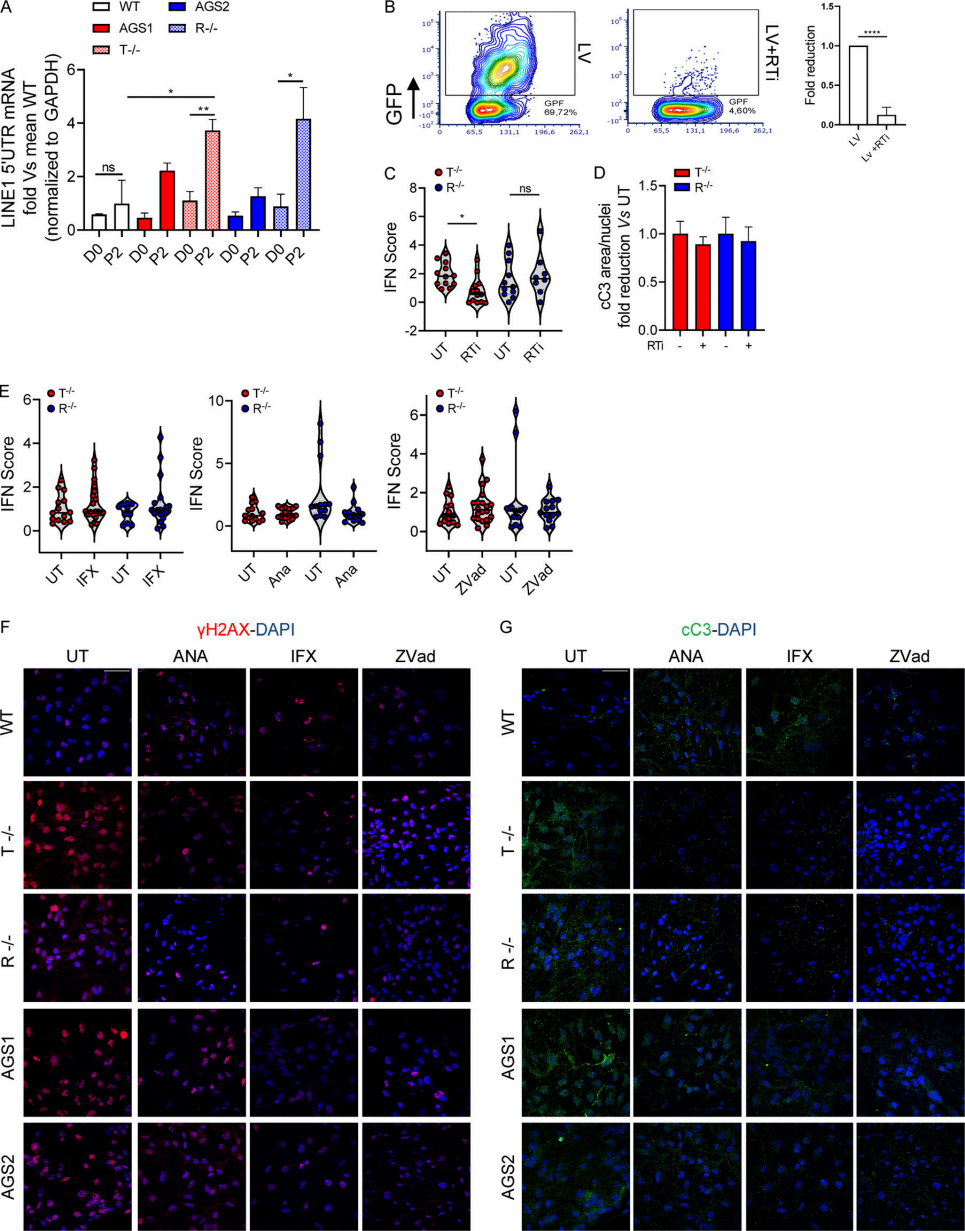
**Impact of the different treatments on KO astrocyte inflammatory profiles. (A)** Line 1 (5′ UTR) mRNA expression levels were measured at different time points during differentiation protocol in WT, KO, and AGS patient–derived cells, expressed as fold difference vs. WT and normalized to the GAPDH housekeeping gene. (Mean ± SEM; *n* = 3 independent experiments; one-tailed Mann–Whitney *U* test; *, P < 0.05; **, P < 0.01.) **(B)** Representative FACS plot and fold reduction quantification of GFP + iPSC-derived astrocytes 5 d after lentiviral vector transduction at MOI 10 with and without the RTi cocktail. (Mean ± SD; *n* = 2 independent experiments; one-tailed Mann–Whitney *U* test; ****, P < 0.0001.) **(C)** Violin plots showing the distribution of IFN scores calculated from the median FC of six ISGs in T^−/−^ and R^−/−^ KO cells treated with RTi (*n* = 3 independent experiments: Wilcoxon rank-sum test; *, P < 0.05). **(D)** WT neuron expression of cleaved Caspase-3 (cC3) upon 48-h exposure to KO iPSC–derived astrocyte conditioned medium treated with the RTi cocktail measured by IF staining. Quantification of two slides of two independent differentiations. (Mean ± SD; *n* = 2 independent experiments.) **(E)** Violin plots showing the distribution of IFN scores calculated from the median FC of six ISGs in T^−/−^ and R^−/−^ KO cells treated with infliximab (IFX), anakinra (ANA), or Zvad. (*n* = 5–6 independent experiments.) **(F and G)** Representative images of WT neurons treated or not with conditioned medium collected from AGS patient–derived and KO astrocytes ± ANA, IFX, or Zvad. One image per genotype per condition shown. Scale bar, 50 µm.

RT of EREs has been suggested as a leading cause of neuroinflammation in AGS (Thomas et al., 2017). Accordingly, we observed higher expression of LINE1 in KO cells during the differentiation process from iPSC-NSCs toward astrocytes compared with their WT counterparts (Fig. S5 A). However, blocking LINE1 RT with a previously reported RT inhibitor (RTi) cocktail composed of 3TC and d4T (Thomas et al., 2017), which efficiently blocks RT in iPSC-derived astrocytes (Fig. S5 B), reduced the IFN score only in TREX1-deficient iPSC-derived astrocytes, whereas it had no impact on RNASEH2B KO cells (Fig. S5 C). In addition, conditioned medium from KO iPSC–derived astrocytes treated with RTi did not prevent neurotoxicity (Fig. S5 D), suggesting the involvement of additional triggers independent of ERE RT that contribute to neurotoxicity in both TREX1- and RNASEH2B-deficient contexts.

Given the extent of DNA damage observed and its contribution to the IFN scores in both TREX1- and RNASEH2B-deficient astrocytes, we sought to further dissect its role in AGS neuroinflammation. Inhibiting p53 has been shown to rescue RNASEH2B-asociated proliferation defects in primary cultures of embryonic mesodermal tissue (Reijns et al., 2012) and to prevent neurotoxicity in a murine model of neural Rnaseh2b inactivation (Aditi et al., 2021). To test whether p53 inhibition could rescue AGS-associated inflammation, we transduced WT and KO iPSC–derived astrocytes with a lentiviral vector constitutively expressing the dominant-negative p53-inhibiting peptide GSE56 (Piras et al., 2017) and assessed their neurotoxic potential 72 h after transduction. Whereas GSE56 expression slightly increased the IFN scores of AGS astrocytes (Fig. S4 F), conditioned medium from these astrocytes in which p53-mediated responses were prevented (Fig. S4 G) completely abolished neurotoxicity (Fig. 7 D). This suggests that p53-dependent responses to DNA damage contribute to activating neurotoxic signaling in AGS astrocytes. TREX1- and RNASEH2B-deficient astrocyte secretomes contained several mediators of the Complement cascade (Fig. 6, E and F). Complement has been shown to mediate neurotoxicity in several neurological diseases including Alzheimer’s disease (Guttikonda et al., 2021) and to contribute to activation of proinflammatory pathways in astrocytes (Guttenplan et al., 2021). To test the potential contribution of Complement proteins secreted by the KO astrocytes to neurotoxicity, we exposed WT neurons to conditioned medium from KO astrocytes in the presence or absence of eculizumab, a clinically approved Complement C5 inhibitor. C5 inhibition led to reduced γH2AX foci in these neurons, supporting the contribution of the Complement pathway in AGS neurotoxicity (Fig. 7 D).

We next sought to pharmacologically inhibit key proinflammatory pathways detected in the isogenic KO astrocytes (Fig. 2 B) and in patient-derived cells (Fig. 4, D, G, and H). We exposed the KO and patient iPSC–derived astrocytes to infliximab to inhibit the TNFα cascade, anakinra to block IL1β-mediated inflammation, or Z-Vad, a pan-caspase inhibitor. Remarkably, although these compounds did not reduce the IFN scores in the KO iPSC–derived astrocytes (Fig. S5 E), the conditioned medium collected from the drug-treated iPSC-derived astrocytes completely lost its neurotoxic potential, as measured by H2AX histone phosphorylation and cleaved Caspase-3 accumulation (Fig. 7 E; and Fig. S5, F and G). This suggests that proinflammatory mediators secreted by AGS astrocytes drive neurotoxicity independently of the type I IFN signatures observed in AGS astrocytes. Although we did not detect significant amounts of cytokines secreted by AGS astrocytes (Fig. 6 B), neurotoxicity was significantly reduced when neutralizing antibodies targeting TNFα, IL1β, or IL-8 were added directly on neurons together with conditioned medium from TREX1 or RNASEH2B KO astrocytes (Fig. 7 F), confirming the direct contribution of these proinflammatory cytokines to AGS astrocyte-mediated neurotoxicity.

Taken together, our results identify DNA damage as a key activator of neurotoxic proinflammatory programs in AGS astrocytes that are independent from their type I IFN signatures and provide a framework for the development of effective pharmacological strategies to prevent or counteract AGS in the human CNS.

## Discussion

In this work, we have generated human iPSC-based AGS disease models for RNASEH2B and TREX1 deficiencies that have allowed us to identify DNA damage as a major driver of proinflammatory responses in AGS astrocytes that lead to neurotoxicity, which we were able to prevent pharmacologically. Spontaneous activation of type I IFN and proinflammatory programs observed in astrocytes, but not iPSC-NSCs or neurons, when differentiated separately is in agreement with their proposed role in AGS pathology, as they secrete IFN in the CNS (Sase et al., 2018) and proinflammatory cytokines such as CXCL10 and CCL2 upon transient silencing of AGS genes (Cuadrado et al., 2015; van Heteren et al., 2008), and mediate neurotoxicity in an in vitro iPSC-based model of TREX1 deficiency (Thomas et al., 2017). Our results suggest that the endogenous triggers of AGS may appear later during glial lineage commitment. In agreement, many cases of AGS develop after birth (Crow et al., 2014), in a period of active postnatal brain development (Menassa and Gomez-Nicola, 2018). On the other hand, phenotypes are known to be variable and likely depend on the gene affected or the model used. For example, induction of IFN-stimulated genes (ISGs) has been reported for ADAR1 KO cells when transitioning from embryonic stem cells to neuronal progenitor cells (Chung et al., 2018), and neural inactivation of Rnaseh2b leads to neuropathology in mice (Aditi et al., 2021). Notably, in the context of mixed cultures of AGS patient iPSC–derived CNS cells, all subsets harbored significant transcriptional alterations, involving not only type I IFN and inflammatory pathways, but also significant upregulation of p53-mediated DNA damage and apoptotic responses as well as metabolic alterations, suggesting that active cross talk between astroglial and neural components contributes to AGS physiopathology. Additional experiments on patient-derived neurons differentiated separately with specific protocols will further clarify the central role of the astroglial component in AGS.

The involvement of EREs in AGS has been supported by the observation that LINE1 loci are hypomethylated in patient-derived fibroblasts (Lim et al., 2015), and extrachromosomal accumulation of LINE1 has been reported to drive type I IFN activation in TREX1-deficient astrocytes that could be rescued by blocking LINE1 RT with a cocktail of RTis (Thomas et al., 2017). RTis have also been tested in the context of a recent clinical trial performed in 11 AGS children with mutations in TREX1, SAMHD1, RNASEH2A, and RNASEH2B (Rice et al., 2018). Although a decrease in the type I IFN signature in peripheral blood and CSF was observed for the duration of the RTi administration, this treatment was proposed to be used in combination with other therapies. Moreover, the decay of the type I IFN signature is often associated also with the natural course of the disease (Rice et al., 2012), rendering it more difficult to assess the efficacy of experimental treatments, and effects of RT inhibition on peripheral ISG signatures in healthy individuals have not been reported. In our hands, LINE1 expression was increased upon iPSC-derived astrocyte differentiation in all clones, with KO clones expressing two- to fourfold more LINE1 than WT. However, treatment with RTis lowered the IFN score only in the TREX1-deficient cells, suggesting the involvement of additional endogenous triggers in AGS. In agreement, TREX1 deficiency has been shown to cause systemic autoimmunity despite the presence of antiretroviral drugs in TREX1^−/−^ mice (Achleitner et al., 2017). It is also possible that instead of type I IFN activation, increased ERE expression could lead to a more proinflammatory response in the human CNS, similarly to what has recently been reported in the context of mice deficient for the transcriptional repressor TRIM28 (Jönsson et al., 2021).

In addition, although the type I IFN signature is one of the typical hallmarks of AGS, to the best of our knowledge, direct evidence for a causal link with human disease pathogenesis is lacking. While all AGS iPSC–derived astrocytes harbored active type I IFN and proinflammatory signatures at steady state, the contribution of the type I IFN–independent inflammatory cascades seems more prominent in terms of consequent neurotoxicity for both TREX1- and RNASEH2B-deficient cells, as drugs abrogating neurotoxicity failed to reduce the IFN scores in KO astrocytes. The spontaneous activation of DNA damage and inflammatory pathways in RNASEH2B- and TREX1-deficient cells of the human CNS is in line with detection of markers of inflammatory pathways involving NF-κB and the inflammasome complex in patient serum and CSF (Crow and Livingston, 2008; Takanohashi et al., 2013). Our data indicate that increased DNA damage activates proinflammatory responses in AGS astrocytes across genotypes. Accordingly, accumulation of R-loops and micronuclei containing damaged DNA was detected in both TREX1- and RNASEH2B-defective iPSC-derived astrocytes. Accumulation of micronuclei was recently associated with type I IFN activation in TREX1-deficient cells (Mohr et al., 2021), RNase H2-deficient mice, and murine cells (Bartsch et al., 2017; Mackenzie et al., 2016; Mackenzie et al., 2017). They are also among the most widely studied biomarkers of DNA damage and chromosomal instability in humans (Fenech et al., 2020). Moreover, micronuclei are associated with activation of proinflammatory pathways with inflammation triggering DNA damage and consequently micronuclei formation, leading to a vicious cycle that has been suggested to contribute to several autoimmune diseases (Kirsch-Volders et al., 2020).

R-loops can act as a major source of DNA damage due to the fragility of the exposed ssDNA during transcription or by impeding DNA replication in the S phase of the cell cycle (Crossley et al., 2019; García-Muse and Aguilera, 2019). RNA/DNA hybrids themselves have been shown to activate various innate immune sensors (cGAS, TLR9, and the NLPR3 inflammasome) in macrophages and dendritic cells, resulting in inflammatory responses (Kailasan Vanaja et al., 2014; Mankan et al., 2014; Rigby et al., 2014). In addition, R-loops have been very recently shown to trigger the release of cytoplasmic ssDNAs, leading to chronic inflammation upon DNA damage (Chatzidoukaki et al., 2021). These data are in line with the observed contribution of both STING and NLRP3 to the increased IFN scores in AGS astrocytes and raise the possibility that RNA/DNA hybrids provide a relevant link between DNA damage and innate immune activation in AGS. While the link between RNASEH2B deficiency and increased R-loop formation is consistent with the ability of the RNase H2 complex to degrade the RNA strand of RNA/DNA heteroduplexes, the connection between R-loop homeostasis and TREX1 is less evident, as the cytosolic DNA exonuclease has mainly been implicated in degradation of ssDNA and dsDNA in the context of viral infections such as HIV (Yan et al., 2010) or upon RT of the ERE LINE-1 (Stetson et al., 2008b). Of note, TREX1 has been shown to translocate into the nucleus upon DNA damage and to interact with PARP1, a nuclear enzyme involved in the DNA damage response (Miyazaki et al., 2014). Interestingly, PARP1 has been recently implicated in preventing R-loop–associated DNA damage through PARylation of TonEBP (Ye et al., 2021), and PARP1 interacts with RNA/DNA helicase DHX9 and R-loops in vivo, preventing R-loop–associated DNA damage (Cristini et al., 2018). It will be of interest to explore if there is a direct link between loss of TREX1 and R-loop homeostasis.

Widespread genome instability is an important consequence of RNase H2 deficiency and the cause of embryonic lethality in mice (Hiller et al., 2012; Reijns et al., 2012). This is largely driven by a failure to remove genome-embedded ribonucleotides (Uehara et al., 2018), with topoisomerase cleavage at such ribonucleotides responsible for most of the observed DNA damage (Zimmermann et al., 2018). Partial-loss-of-function mutations in RNase H2 were also reported to lead to accumulation of embedded ribonucleotides, and this was associated with DNA damage and senescence in systemic lupus erythematosus and AGS patient fibroblasts (Günther et al., 2014). Here, we did not detect increased levels of embedded ribonucleotides in either AGS2 or KO cells with a much more pronounced loss of RNase H2 activity. This lack of changes in genomic ribonucleotide content is likely due to the limit of detection of the alkaline gel assay used, but we cannot exclude cell type–specific differences in ribonucleotide incorporation or the requirement for RNase H2 in their removal. It was previously shown that neither DNA damage nor ISG upregulation in *Rnaseh2b*^−/−^ MEFs was rescued by overexpression of RNase H1, which provided physiological levels of RNase H activity in the cells (Mackenzie et al., 2016). In line with this, endogenous RNase H1 activity was unable to compensate for the loss of RNase H2 and suppress accumulation of R-loops in the *RNASEH2B*-deficient cells. This suggests that these hybrids/R-loops may specifically be degraded by RNase H2 and contribute to the AGS phenotype.

Interestingly, several metabolic pathways including hypoxia, glycolysis and mTORC1 signaling were significantly altered in all patient-derived CNS cell subsets, suggesting that mitochondrial and autophagic dysfunctions may be associated with AGS. Mitochondrial DNA (mtDNA) has been observed to activate proinflammatory responses in various diseases and pathological states, including AGS (Mustelin et al., 2019; Tumienė et al., 2017; West, 2017). Of note, TNFα, which we show to actively contribute to AGS astrocyte-mediated neurotoxicity, has very recently been shown to trigger mtDNA release and cGAS/STING-dependent IFN responses in inflammatory arthritis (Willemsen et al., 2021). It will be of interest to explore the contribution of mtDNA to AGS in our human CNS models of disease. In addition, micronuclei associated with RNase H2 deficiency have been linked to autophagy in mice, as pharmacological mammalian target of rapamacyn (mTOR) inhibition resulted in a significant reduction of cytosolic DNA and the accompanying IFN signature (Bartsch et al., 2017). It is possible that some of these pathways are activated in a compensatory effort of the cells to remove excess of NAs.

Encouraging results are emerging with JAK inhibitors in several distinct type I interferonopathies (Frémond et al., 2016; Sanchez et al., 2018). TREX1-related skin disease and IFIH1-determined systemic inflammation have shown improvements with the JAK1/2 inhibitor, ruxolitinib (Briand et al., 2019; McLellan et al., 2018). Recent results from a clinical trial using baricitinib, an oral JAK1 and JAK2 inhibitor, to treat AGS patients also suggest improved IFN scores (Vanderver et al., 2020). However, not all benefits of these drugs may relate to an inhibition of IFN signaling, given that they have effects beyond JAK1 inhibition at the type I IFN receptor. Moreover, the onset of AGS was reported in a patient with biallelic mutations in *RNASEH2B*, despite the use of ruxolitinib for 10 mo while the patient was presymptomatic (Neven et al., 2020). Our data suggest that type I IFN pathways may not be the major driver of disease in AGS with a relevant contribution of DNA damage-associated apoptotic and proinflammatory responses. In agreement, both TREX1 and RNASEH2B deficiencies have been clinically associated with inflammatory symptoms such as inflammatory myopathy (Tumiene et al., 2017) and systemic inflammation (He et al., 2021). Although links between JAK-STAT signaling, type I IFN induction, and neurotoxicity warrant further investigation in our experimental system through pharmacological inhibitors such as ruxolitinib, IFNα receptor blocking antibodies, or genetic modifications of key players, our observations are in line with a recent report in which type I IFN–independent genomic instability was shown to cause AGS-like cell toxicity in a murine model of neural Rnaseh2b inactivation (Aditi et al., 2021). We show that the spontaneous DNA damage–driven proinflammatory activation of AGS iPSC–derived astrocytes leads to the secretion of proteins with neurotoxic potential such as members of the Complement cascade. Complement has been implicated in several neuroinflammatory diseases including Alzheimer’s disease (Guttenplan et al., 2021) and linked with NLRP3 inflammasome activation (Laudisi et al., 2013). Importantly, AGS astrocyte-mediated neurotoxicity was fully rescued by clinically viable inhibitors of critical mediators of apoptosis and inflammation such as the IL-1βR receptor antagonist anakinra, the TNFα antagonist infliximab, and the Complement inhibitor eculizumab. These results highlight that AGS neurotoxicity cannot be ascribed to a single pathway but is the result of a complex DNA damage–driven proinflammatory cascade involving several mediators that we show to be clinically targetable to block or dampen the harmful neurotoxic effects of the inflammatory activation in AGS astrocytes. In addition, our observations suggest that implementing additional gene signatures to the currently used type I IFN score may allow more accurate monitoring of AGS disease and efficacy of therapeutic strategies in the future.

In summary, we have generated human CNS models of AGS physiopathology that recapitulate several features of the disease and have allowed us to highlight the key role of astrocytes in AGS neurotoxicity. We identify DNA damage as a key activator of neurotoxic proinflammatory programs in AGS astrocytes that are independent from their type I IFN signatures. Finally, we propose a previously unappreciated role of R-loops in AGS neuroinflammation and identify therapeutically targetable common drivers of disease across genotypes, of potential relevance also for other neuroinflammatory disorders.

## Materials and methods

### iPSCs

Healthy donor iPSCs were kindly provided by Angela Gritti (SR-Tiget, Milan, Italy) and previously characterized (clone ND1.3; Meneghini, 2017). Patient-derived iPSCs were kindly provided by Silvia Giliani (University of Brescia, Azienda Socio Sanitaria Territoriale Spedali Civili, Brescia, Italy) and elsewhere characterized (Ferraro et al., 2019a; Ferraro et al., 2019b). All iPSCs were maintained on mitomycin-C–treated MEFs in iPSCM: DMEM/F12 (Gibco), 0.18% sodium bicarbonate 7.5% (Life Technologies), 20% KO serum replacement (Life Technologies), 0.1 mM nonessential amino acids, 1 mM sodium pyruvate, 0.1 mM β-mercaptoethanol (Life Technologies), 2 mM L-glutamine, 100 U/ml penicillin/streptomycin, and 10 ng/ml FGF2. iPSC culture was split using collagenase IV (Gibco) at ratios of 1:3 to 1:6. Human cells were used according to the guidelines on human research issued by the San Raffaele Scientific Institute Ethic Committee (protocol TIGET-HPCT).

### iPSCs KO generation

iPSCs were detached with 1 ml Accutase and collected in a 15-ml Falcon tube with 1 ml medium to inactivate the enzyme. Cells were then centrifuged at 200 *g* for 5 min and resuspended in 1 ml iPSCM in the presence of 10 μM of the Rho kinase inhibitor Y-27632 (Sigma-Aldrich). Cells were plated on 0.1% gelatin-coated wells (Sigma-Aldrich) for 1 h to allow MEFs to attach. Floating iPSCs were collected and plated in iPSCM in the presence of 10 μM of the Rho kinase inhibitor Y-27632 on Matrigel-coated dishes. When iPSCs reached 50–70% confluence, cells were detached with 1 ml Accutase and collected in a 15-ml Falcon tube with 1 ml medium to inactivate the enzyme. Cells were then centrifuged at 200 *g* for 5 min and resuspended in iPSCM to be counted. 2 × 10^5^ cells per condition were centrifuged again and resuspended in 17 μl per condition of P3-supplemented nucleofection buffer (Lonza). The mix containing plasmids expressing guide RNA (gRNA) and Cas9 was then added, final volume 20 μl, and cells were immediately nucleofected with AMAXA 4D-nucleofector (Lonza), program CB 150. Immediately after nucleofection, 100 μl medium was added, and cuvettes were stored at 37° for 5–10 min. Nucleofected cells were then collected and diluted in 5 ml medium to be plated dropwise in a 15-cm MEF-coated plate in iPSCM in the presence of 10 μM of the Rho kinase inhibitor Y-27632. Cells were checked daily. On day 7 after nucleofection, >20 colonies/condition were picked and plated, each clone in 1 well of a 96 multiwell (96MW). Meanwhile, ∼30–40 colonies were picked and pulled together to evaluate the overall efficiency. On day 12 after nucleofection, colonies in the 96MW were split in two: one half was replated in MEF-coated 48MW and the other half in Matrigel-coated 48MW. Matrigel colonies were used for nonhomologous end joining assay screening and MEF colonies for amplification and cryopreservation (20 vials/gRNA; 1 vial/well).

### gRNA design

gRNAs targeting the first two conserved coding exons of the RNASEH2B and TREX1 human genes were designed with the CHOPCHOP online tool (https://chopchop.cbu.uib.no/about). The protospacer sequence of the gRNAs used to knock out RNASEH2B is 5′-GTC​CCC​GCA​GTC​CAC​GCC​AG-3′, and the protospacer sequence of the TREX1 guide is 5′-GCT​CAG​ACC​TGT​GAT​CTC​GC-3′. They were assembled from single-strand oligonucleotides ordered from Sigma-Aldrich and cloned in a plasmid expressing them as fusion transcripts with the previously described tracrRNA^(F+E)^ from the human U6 promoter.

### Neural differentiation of iPSCs

For neural induction, hiPSC colonies were detached with Accutase (Life Technologies), suspended as single cells, and plated on 0.1% gelatin-coated wells (Sigma-Aldrich) for 1 h to allow MEFs to attach. Floating iPSCs were then collected and plated on Matrigel (BD Pharmingen)-coated dishes (50,000 cells/cm^2^) in MEF preconditioned iPSCM in the presence of 10 μM of the Rho kinase inhibitor Y-27632. When the cell culture reached ≈90% confluence (usually 2 d after plating), the culture medium was replaced with KO serum replacement medium supplemented with 200 ng/ml of rhNOGGIN (R&D) and 10 μM of SB431542 (Sigma-Aldrich). The medium was changed daily for the next 3 d. Thereafter, it was switched every other day to gradually expose the cells to increasing (1:3, 1:1, 3:1) ratios of NSC/KO serum replacement medium. 2 d after the final switch, the cells were detached using Accutase and plated on Matrigel-coated dishes in neural stem cell medium (NSCM) supplemented with 20 ng/ml basic fibroblast growth factor and 20 ng/ml EGF in the presence of 10 μM of the Rho kinase inhibitor Y-27632, according to the published Dual Smad inhibition protocol (Chambers et al., 2009).

### Differentiation of iPSC-NSCs into mixed neuronal/glial cultures and proinflammatory astrocytes

Cells were detached using Accutase and plated on Matrigel-coated dishes (20,000 cells/cm^2^) in NSCM (day 0). During the first 4 d after plating (d4), we gradually replaced NSCM containing basic fibroblast growth factor and EGF with an increasing amount of glial differentiation medium, supplemented with 10 ng/ml platelet-derived growth factor AA, 10 ng/ml NT3, 10 ng/ml IGF-1, 5 ng/ml hepatocyte growth factor, and 60 ng/ml T3. From day 4 to day 13, medium was changed every other day. From day 14 to day 34, the medium was switched to the one described in Santos et al. (2017), supplemented with 10% FBS and 10 ng/ml leukemia inhibitory factor (LIF; Frati et al., 2018). The mixed neural culture obtained on d34 was used for scRNA-seq experiments. For proinflammatory astrocyte enrichment, the d34 neural mixed culture was detached with Accutase and replated (passage 0) in the same medium to deplete neurons and oligodendrocytes through mechanical dissociation and detach/replate steps. Astrocytes were kept in culture for a maximum of three passages. Drug exposure (for concentrations, see Table S4) was performed for 24 h on passage 1 or 2 astrocytes plated at 1 × 10^5^ cells/well in a 24MW plate. Transduction was performed on passage 1 or 2 astrocytes plated at 1 × 10^5^ cells/well in a 24MW plate at MOI 10 with the GSE56-overexpressing vector previously described (Piras et al., 2017) or a GFP-overexpressing vector as control.

### Differentiation of iPSC-NSCs into iPSC-derived neurons

iPSC-NSCs obtained through the Dual Smad inhibition protocol, as described above, were further differentiated into neurons with the STEMdiff Neurons Differentiation Kit and maintained in culture with the STEMdiff Neuron Maturation kit following manufacturer’s instructions. Cells were analyzed and used for experiments after 21 d of maturation. On day 15 of differentiation, conditioned medium from KO or AGS astrocytes (at steady state or upon drug treatment) mixed with neuronal medium (ratio 2:1) was added on neurons for 48 h and removed just before paraformaldehyde cell fixing. For neutralizing antibody rescue, conditioned medium from KO or AGS astrocytes at steady state mixed with neuronal medium (ratio 2:1) supplemented with infliximab, anti-IL8, or eculizumab (for concentrations, see Table S4) was added to neurons for 48 h and removed just before paraformaldehyde cell fixing.

### Gene expression

Total RNA was extracted with the RNeasy Plus Micro kit or RNeasy Micro Kit (Qiagen), and RT was performed using SuperScriptVILO cDNA Synthesis Kit (Thermo Fisher Scientific) according to manufacturer’s instructions. Gene expression analysis was performed by Taqman assay as previously described. Human HPRT1 was used to normalize the total quantity of human cDNA input. Refer to Table S1 for the complete list of Taqman probe reagents. LINE1 5′ UTR mRNA levels were measured by real-time PCR (Conti et al., 2015) and normalized on GAPDH housekeeping expression; the primers used are shown in Table S2.

### Molecular analyses

For molecular analyses, genomic DNA was isolated with DNeasy Blood & Tissue Kit or QIAamp DNA Micro Kit (Qiagen). KO efficiency was measured by mismatch-sensitive endonuclease assay by PCR-based amplification of the targeted locus followed by digestion with Surveyor Mutation Detection Kit according to the manufacturer’s instructions. Because the Surveyor Nuclease cuts DNA at sites of duplex distortions, the products of re-annealing between WT and mutant alleles (carrying mutations or deletions consequent to CRISPR/Cas9 activity) are specifically digested. Digested DNA fragments were resolved and quantified by capillary electrophoresis on Spreadex gel (Duotech) according to the manufacturer’s instructions.

### Sequencing of the CRISPR-target sequences

To assess the outcome of RNASEH2B and TREX1 DNA repair upon delivery of the CRISPR/Cas9 nuclease system, the sequence surrounding the CRISPR cut site was amplified by PCR. Individual PCR products were purified from gel using the Wizard gel purification kit (Promega) and cloned into plasmid vectors using the TOPO-TA Cloning kit (Invitrogen), according to the manufacturer’s instructions. Sanger DNA sequencing from 10 minipreps per TOPO-TA cloning was then performed at GATC (Eurofins-Genomics).

### RNase H2 enzymatic activity assay

To assess RNase H2 activity in whole-cell extracts, a fluorescence resonance energy transfer–based fluorescent substrate release assay was performed as previously described (Reijns et al., 2012). Briefly, RNase H2-specific activity was determined by measuring the cleavage of a single-embedded ribonucleotide-containing dsDNA substrate (DRD:DNA). Activity against a DNA:DNA substrate of the same sequence was used to correct for non-RNase H2 background activity against the DRD:DNA substrate. Substrates were formed by annealing a 3′-fluorescein-labeled oligonucleotide (5′-GAT​CTG​AGC​CTG​GGa​GCT-3′ or 5′-GAT​CTG​AGC​CTG​GGA​GCT-3′; uppercase DNA, lowercase RNA) to a complementary 5′-DABCYL-labeled DNA oligonucleotide (Eurogentec). Reactions were performed in 100 μl of reaction buffer (60 mM KCl, 50 mM Tris-HCl, pH 8.0, 10 mM MgCl_2_, 0.01% BSA, and 0.01% Triton X-100) with 250 nM substrate in 96-well flat-bottomed plates at 24°C. Whole-cell lysates were prepared as described (Benitez-Guijarro et al., 2018), and the final protein concentration used per reaction was 100 ng/μl. Fluorescence was read (100 ms) every 5 min for ≤90 min using a VICTOR2 1420 multilabel counter (PerkinElmer) with a 480-nm excitation filter and a 535-nm emission filter.

### Detection of ribonucleotides in genomic DNA

To determine the presence of excess genome-embedded ribonucleotides in nuclear DNA, alkaline gel electrophoresis of RNase H2–treated genomic DNA was performed as previously described (Benitez-Guijarro et al., 2018). Briefly, total NAs were isolated from pellets from ∼1 million cells (after whole-cell lysate preparation for the RNase H2 enzymatic activity assay) by incubation in ice-cold buffer (20 mM Tris-HCl, pH 7.5, 75 mM NaCl, and 50 mM EDTA) with 200 µg/ml proteinase K (Roche) for 10 min on ice, followed by addition of *N*-lauroylsarcosine sodium salt (Sigma-Aldrich) to a final concentration of 1%. Nucleic acids were phenol:chloroform extracted, isopropanol precipitated, and dissolved in nuclease-free water. For alkaline gel electrophoresis, 500 ng total NAs was incubated with 1 pmol purified recombinant human RNase H2 (isolated as previously described [Reijns et al., 2011]) and 0.25 µg of DNase-free RNase (Roche) for 30 min at 37 °C in 100 μl reaction buffer (60 mM KCl, 50 mM Tris-HCl, pH 8.0, 10 mM MgCl_2_, and 0.01% Triton X-100). Nucleic acids were ethanol precipitated and dissolved in nuclease-free water, and 250 ng was separated on 0.7% agarose gels in 50 mM NaOH and 1 mM EDTA. After overnight electrophoresis, the gel was neutralized in 0.7 M Tris-HCl, pH 8.0, and 1.5 M NaCl and stained with SYBR Gold (Invitrogen). Imaging was performed on a FLA-5100 imaging system (Fujifilm), and densitometry plots were generated using AIDA Image Analyzer (Raytest).

### Western blot (WB)

WB was performed as previously described (Kajaste-Rudnitski et al., 2011; Kajaste-Rudnitski et al., 2006). Samples were subjected to SDS-PAGE on Bolt 4–12% Bis-Tris Plus gels (Thermo Fisher Scientific, NW04120BOX) and transferred to polyvinylidene difluoride membrane by electroblotting. Refer to Table S3 for the list of antibodies and dilutions. In WB quantifications, signal was quantified by densitometry using ImageJ software and normalized to actin, Histone H3, or GAPDH then log10 transformed for the purposes of the correlation statistics and graphical representation when needed.

### Immunofluorescence (IF)

Cells were fixed with 4% paraformaldehyde (in 1× PBS) for 20 min at room temperature and permeabilized with 0.1% Triton X-100 for 20 min at room temperature. For blocking nonspecific sites, cells were incubated for 30 min in PBS + 10% next generation sequencing and stained overnight at 4°C with primary antibodies (refer to Table S3 for the complete list of antibodies and dilutions). After three washes with 1× PBS, cells were incubated with donkey anti-rabbit IgG, Alexa Fluor 488 (1:500 dilution; A-21206; Thermo Fisher Scientific) or donkey anti-mouse IgG, Alexa Fluor 555 (1:500 dilution; A-31570; Thermo Fisher Scientific) for 2 h at room temperature. Nuclei were stained with DAPI (10236276001, Roche) for 10 min at room temperature.

### Image acquisition and analysis

Images were recorded using the TCS SP5 Leica confocal microscope, 60× with oil, by Advanced Light and Electron Microscopy Bioimaging Center facility or were visualized with Nikon Eclipse Ni microscope using double-laser microscopy with a Plan-Fluor (20× air and 40× air magnification) objective lens (Nikon). Images were acquired using a camera (Nikon DS Fi2) and NIS-Element F4.00.00 acquisition software (Nikon). The immunopositive area of acquired images (expressed in pixels) was calculated using ImageJ or Cell profiler software. ND samples were used to set the threshold. The immunopositive area was normalized to the number of nuclei in the same field. At least five fields/sample with 50–70 cells/field were quantified (∼300 cells per staining were quantified). Cellular covers stained with anti-phospho histone γH2AX (Ser139) primary antibody and with DAPI, were used for Micronuclei quantification. More than 700 nuclei were analyzed for KO samples, and more than 1,400 were analyzed for WT and patient-derived cells. Image analysis was performed using Image J and Cell Profiler software. In particular, images were zoomed in and fluorescent signals were enhanced to detect and quantify micronuclei. Image quantification shows the percentage of micronuclei-positive cells or the percentage of γH2AX-positive micronuclei calculated on the total number of cells per slide.

### TP53 sequencing

iPSC WT and KO DNA extraction was performed with DNeasy Blood & Tissue Kit or QIAamp DNA Micro Kit (Qiagen). The libraries were prepared with the amplicon-based VariantPlex TP53 (ArcherDX) kit, and sequencing was performed on a MiSeq v2 Micro flowcell in paired-end mode (2 × 150 bp). Raw reads were quality controlled with FastQC (http://www.bioinformatics.babraham.ac.uk/projects/fastqc, v0.11.6) and trimmed with Trimmomatic (Bolger et al., 2014) v0.39 in paired-end mode, setting the quality threshold equal to 20 and the minimum length equal to 25. High-quality reads were then aligned with BWA-MEM (Li and Durbin, 2010) v0.7.17-r1188 using GRCh38.p13 as reference genome. Alignments were sorted and indexed using Picard tools (https://broadinstitute.github.io/picard, v2.22.3). The variant call analysis workflow was performed according to the Genome Analysis Tool Kit guidelines (McKenna et al., 2010) v4.2.0.0; in particular, base quality score recalibration was performed using the BaseRecalibrator, ApplyBQSR, and BaseRecalibrator modules; the variants were called and filtered, respectively, with Genome Analysis Tool Kit Mutect2 and FilterMutectCalls modules. The variant call files were filtered to retain only variants classified as “PASS” and were loaded into the R environment (v4.0.3) with the readVcf function from the R/Bioconductor package VariantAnnotation (v1.32.0). The variants were visualized using the lolliplot function from the R/Bioconductor package trackViewer (v1.22.0).

### RNA-seq and analysis

Quality of raw single-end reads was determined using FastQC, and read trimming was performed using Trim_galore to remove residual adapters and low-quality sequences. Trimmed reads were aligned against the human reference genome (GRCh38, p13) using STAR (Dobin et al., 2013) v2.7.6a with standard input parameters, and only uniquely mapped reads were considered for downstream analyses. Reads were assigned to genes with featureCounts (Liao et al., 2014) v2.0.1, using the GENCODE primary assembly v34 gene transfer file as reference annotation for the genomic features. Transcript count matrices were then imported into the R statistical environment and processed by the R/Bioconductor package Deseq2 (Love et al., 2014) following the standard workflow. Genes with adjusted P values <0.1 were considered as differentially expressed.

Functional enrichment analysis was performed on lists of differentially expressed genes. GSEA was performed considering different datasets (Gene Ontology, Kyoto Encyclopedia of Genes and Genomes Pathway Database, Reactome Pathway Database, Molecular Signatures Database) using the R/Bioconductor package clusterProfiler (Yu et al., 2012) v3.18, by preranking genes according to log2(fold-change [FC]) values. Enrichment P values were corrected for multiple testing using false discovery rate and considered statistically significant if <0.05. Volcano plots were generated using the R package ggplot2 (v3.3.4) and used to display RNA-seq results, plotting the statistical significance (P value) vs. the magnitude of change (FC). Heatmaps were generated using the R package pheatmap (v1.0.12).

### IFN and inflammation scores analysis

IFN scores derived from the sequencing expression data (scRNA-seq data of AGS patients and RNA-seq data from KO cells) were calculated in 32 selected IFN-related genes (IFN-RGs: IFI27, IFI44L, IFIT1, ISG15, RSAD2, SIGLEC1, IFIT3, SERPING1, IFITM1, IRF7, STAT1, C1QA, CXCL10, EPSTI1, GBP1, HERC5, HERC6, IFI44L, IFIT2, IFI6, IFIT5, LAMP3, OAS1, MX1, OAS2, OAS3, OASL, PLSCR1, RTP4, SOCS1, SPATS2L, and USP18) using the z-score–based standardized IFN score calculation method as reported previously (Kim et al., 2018; Lambers et al., 2019; Rice et al., 2013; Rice et al., 2018; Tungler et al., 2016). In detail, z-scores for each of the 32 genes were calculated using the following equation as described in Kim et al. (2018):

$$z‐\mathrm{score}\;\mathrm{for}\;\mathrm{each}\;\mathrm{gene}=\;\frac{\left[gene\;count-mean\left(WT\;gene\;expression\right)\right]}{SD\left(WT\;gene\;expression\right)}\text{.}$$

As the calculation for each z-score is relative to the mean and SD of the WT samples, each z-score can become negative if the gene expression is below the mean of that in the WT samples. For the WT samples, the scores were calculated dividing the mean expression of each gene by the SD.

Using the same approach and considering the same expression data, inflammation scores were calculated in 30 selected inflammation-related genes (IRGs: CXCL8, CXCL6, SLC7A2, NINJ2, CCR7, IL1B, TLR2, LIF, CCL2, CASP1, IL1R1, TLR3, LPAR1, PTGER2, IL15, CD6, RELA, NFKB1, IGF1R, CARD8, IL4R, IL7R, IRF1, MMP9, IFNGR1, JUNB, SERPINE1, BTG2, IRF9, and PTPN11).

Regarding the expression data from the qRT-PCR experiments, the IFN scores and inflammation scores were calculated from a panel of six ISGs (ISG15, IFI27, IFIT1, RSAD2, USP18, and cGAS) and six IRGs (CXCL8, IL1B, IL6, CARD8, CASP1, and TNFA), as previously described (Rice et al., 2013; Rice et al., 2018; Tungler et al., 2016). The expression of each target gene was normalized against the geometric mean of the housekeeping gene (HPRT1). Relative quantification (RQ) was calculated based on the cycle threshold (Ct) values as follows (Livak and Schmittgen, 2001):

$$RQ=2e^{-\left[Ct\left(targetgene\right)-Ct\left(HPRT1\right)\right]}\text{.}$$

The median FC in the relative mRNA of the selected ISGs and IRGs compared with the median of combined healthy controls was used to calculate the IFN score and inflammation score, respectively, for each sample.

### Generation and analysis of scRNA-seq data

#### Data generation

scRNA-seq libraries were generated using a microfluidics-based approach on Chromium Controller (10x Genomics) using the Chromium Single Cell 3′ Reagent Kit v3.1 according to the manufacturer’s instructions. Briefly, single cells from a neural mixed culture (previously described) were suspended in 0.4% BSA-PBS, at a concentration ranging from 1,000 to 2,000 cells/μl. 8,700 cells were added to each channel to achieve a recovery rate of 5,000 cells per sample. Cells were partitioned in Gel Beads in Emulsion and lysed, followed by RNA barcoding, RT, and PCR amplification (13 cycles). The concentration of the scRNA-seq libraries was determined using Qubit v3.0, and size distribution was assessed using an Agilent 4200 TapeStation system. Libraries were sequenced on an Illumina NovaSeq instrument (paired-end, 150-bp read length).

#### Data processing and graph-based clustering

Raw data from scRNA-seq was analyzed and processed into transcript count matrix by Cell Ranger (https://support.10xgenomics.com/single-cell-gene-expression/software/pipelines/latest/what-is-cell-ranger, v4.0.0) from the Chromium Single Cell Software Suite by 10x Genomics. Fastq files were generated using the Cell Ranger mkfastq command with default parameters. Gene counts for each cell were quantified with the Cell Ranger count command with default parameters. For all analyses, human genome (GRCh38.p13) was used as the reference. The resultant gene expression matrix was imported into the R statistical environment for further analysis.

Cell filtering, data normalization, and clustering were carried out using the R package Seurat (Stuart et al., 2019) v3.2.2. For each cell, the following quality measures were calculated: percentage of mitochondrial genes and number of total genes expressed. Cells with a ratio of mitochondrial vs. endogenous gene expression >0.2 were excluded as putative dying cells. Cells expressing <200 or >8,000 total genes were also discarded as putative poorly informative cells and multiplets. Counts were normalized using Seurat function NormalizeData with default parameters. Expression data were than scaled using the ScaleData function, regressing on number of unique molecular identifier, percentage of mitochondrial gene expression, and difference between S and G2M scores. Cell cycle scores were calculated using the CellCycleScoring function.

The different single-cell datasets were integrated in a single object using the R package Harmony v1.0 approach (Korsunsky et al., 2019) for dealing with experimental and biological confounding factors and removing batch effects. Dimensionality reduction was then performed with principal component analysis on the batch-corrected data. Uniform Manifold Approximation and Projection (UMAP) dimensionality reduction (McInnes et al., 2018) was performed on the calculated principal components to obtain a 2D representation for data visualization. Cell clusters were identified using the Louvain algorithm at resolution *r* = 0.6, implemented by the FindCluster function of Seurat. To characterize each cluster, a comprehensive manual annotation was performed. A list of marker genes for different cell types was collected from a literature-curated set of relevant marker genes (Darmanis et al., 2015; Guttikonda et al., 2021; Jerber et al., 2021; Tanaka et al., 2020; Zhang et al., 2016).

#### Differential expression and GSEA

To find the differentially expressed (marker) genes for the annotated clusters, the functions FindAllMarkers (iteratively comparing one cluster against all the others) and FindMarkers (two condition comparison) from the Seurat package were used with default parameters. Significant differentially expressed genes were identified using the following parameters: adjusted P values <0.05, average log FC >0.25, and percentage of cells with expression >0.1. Downstream analysis, including GSEA, was performed with R/Bioconductor package cClusterProfiler using a list of databases including Gene Ontology, Kyoto Encyclopedia of Genes and Genomes Pathway Database, Reactome Pathway Database, and Molecular Signatures Database. Enriched terms with a *q* value <0.05 were considered statistically significant. Heatmaps were produced using the R package pheatmap. Charts and images were produced using R package ggplot2.

### DRIP

DRIP was performed as described previously (Cristini et al., 2018). Briefly, non-crosslinked nuclei were lysed in nuclear lysis buffer (50 mM Tris-HCl, pH 8.0, 5 mM EDTA, and 1% SDS) and digested with proteinase K (Sigma-Aldrich) at 55°C for 3 h. Genomic NAs were precipitated with isopropanol, washed in 75% ethanol, and sonicated with Bioruptor (Diagenode) in IP dilution buffer (16.7 mM Tris-HCl, pH 8.0, 1.2 mM EDTA, 167 mM NaCl, 0.01% SDS, and 1.1% Triton X-100). Samples were precleared in the presence of protease inhibitors (0.5 mM PMSF, 0.8 mg/ml pepstatin A, and 1 mg/ml leupeptin) with protein A Dynabeads (Invitrogen) blocked with BSA (B8894; Sigma-Aldrich). 10 µg of precleared genomic DNA was incubated overnight at 4°C with S9.6 antibody (Boguslawski et al., 1986) or no antibody. RNase H digestion was performed by incubation with 1.7 U RNase H (NEB, M0297) per µg of genomic DNA for 2.5 h at 37°C before IP. BSA-blocked protein A Dynabeads were added to collect immunocomplexes and washed once with buffer A (20 mM Tris-HCl, pH 8.0, 2 mM EDTA, 0.1% SDS, 1% Triton X-100, and 0.150 M NaCl), once with buffer B (20 mM Tris-HCl, pH 8.0, 2 mM EDTA, 0.1% SDS, 1% Triton X-100, and 0.5 M NaCl), once with buffer C (10 mM Tris-HCl, pH 8.0, 1 mM EDTA, 1% NP-40, 1% sodium deoxycholate, and 0.25 M LiCl), and then twice with buffer D (10 mM Tris-HCl, pH 8.0, and 1 mM EDTA). Elution was performed in 1% SDS and 0.1 M NaHCO_3_. Samples were digested with proteinase K (Sigma-Aldrich) at 45°C for 2 h, and DNA was purified with QIAquick PCR purification kit (Qiagen) and analyzed by qRT-PCR with Rotor-Gene Q and QuantiTect SYBR green (Qiagen). At a certain gene region, the amount of immunoprecipitated material was calculated as the percentage of input after subtracting the background signal (no antibody control). The primers used are shown in Table S2.

### Slot blot

RNA/DNA hybrid slot blot was performed as described (Cristini et al., 2018; Kotsantis et al., 2016). RNase H sensitivity was carried out by incubation with 1.7 U of RNase H (NEB, M0297) per µg of genomic DNA for 2.5 h at 37°C. For loading control, 250 ng of genomic DNA was heated at 95°C for 10 min and loaded on the Slot Blot, and the membrane was denatured in 0.5 M NaOH and 1.5 M NaCl for 5 min and neutralized for 2 min in 0.5 M Tris-HCl, pH 7.2, and 1.5 M NaCl. The membrane was probed with αssDNA (MAB3034; Millipore) after UV crosslinking and saturating. Images were acquired with LAS-4000 (Fujifilm) or by chemiluminescence using autoradiography. S9.6 and ssDNA signals were quantified using Image Studio Lite software (Li-COR Biosciences).

### Secretome and ELISA analysis

The samples were processed using the S-Trap filter (Protifi) according to the manufacturer’s procedure. Briefly, precipitated secretions were solubilized in 50 µl of 5% SDS. Samples were reduced with 10 mM dithiothreitol at 55°C for 30 min, cooled to room temperature, and alkylated with 25 mM iodoacetamide in the dark for 30 min. Next, a final concentration of 1.2% phosphoric acid and then six volumes of binding buffer (90% methanol and 100 mM triethylammonium bicarbonate [TEAB], pH 7.1) were added to each sample. After gentle mixing, the protein solution was loaded to a S-Trap filter and spun at 1,000 *g* for 1 min, and the flow-through was collected and reloaded onto the filter. This step was repeated three times, and then the filter was washed with 200 μl of binding buffer three times. Finally, 1 μg of sequencing-grade trypsin (Promega) and 150 μl of digestion buffer (50 mM TEAB) were added onto the filter, and digestion was carried out at 37°C for 6 h. To elute peptides, three stepwise buffers were applied, with 100 μl of each with one more repeat, including 50 mM TEAB, 0.2% formic acid in H_2_O, and 50% acetonitrile and 0.2% formic acid in H_2_O. The peptide solutions were pooled, lyophilized, and resuspended in 40 µl of 0.1% formaldehyde (FA). 20 µl of each sample was loaded onto individual Evotips for desalting and then washed with 20 μl of 0.1% FA, followed by the addition of 100 μl storage solvent (0.1% FA) to keep the Evotips wet until analysis. The Evosep One system was used to separate peptides on a Pepsep column (150 µm inner diameter, 15 cm) packed with ReproSil C18 1.9 µm, 120 A resin. The system was coupled to the timsTOF Pro mass spectrometer (Bruker Daltonics) via the nano-electrospray ion source (Captive Spray; Bruker Daltonics). The mass spectrometer was operated in parallel accumulation–serial fragmentation mode. The ramp time was set to 100 ms, and 10 parallel accumulation–serial fragmentation MS/MS scans per topN acquisition cycle were acquired. MS and MS/MS spectra were recorded from *m*/*z* 100 to 1,700. The ion mobility was scanned from 0.7 to 1.50 V s^−1^ cm^−2^. Precursors for data-dependent acquisition were isolated within ±1Th and fragmented with an ion mobility–dependent collision energy, which was linearly increased from 20 to 59 eV in positive mode. Low-abundance precursor ions with an intensity above a threshold of 500 counts but below a target value of 20,000 counts were repeatedly scheduled and otherwise dynamically excluded for 0.4 min. Raw data files were converted to peak lists in the MGF format, and downstream identification, validation, filtering, and quantification were managed using FragPipe v13.0. MSFragger v3.0 was used for database searches against a human database with decoys and common contaminants added. The settings were as follows. Identification: trypsin, specific, with a maximum of 2 missed cleavages, ≤2 isotope errors in precursor selection allowed for, 10.0 ppm as MS1, and 20.0 ppm as MS2 tolerances; fixed modifications: carbamidomethylation of C (+57.021464 dalton); variable modifications: oxidation of M (+15.994915 dalton), acetylation of protein N-term (+42.010565 dalton), pyrolidone from peptide N-term Q or C (−17.026549 dalton).

Statistical analysis was conducted using RStudio (v4.0.3), using the R/Bioconductor package DEP (Zhang et al., 2018) v1.12.0. First, proteins were filtered for those identified in at least one technical replicate from each condition. The raw intensities for the remaining proteins were normalized using the variance-stabilizing normalization approach, and imputation of missing data was performed using the MinProb approach, relying on minimal intensity values observed for each sample via the DEP package. As part of the quality control step, all samples were assessed for technical artifacts and outliers using principal component analysis on the top 200 most variable proteins. Differential enrichment analysis was performed relying on linear model. Adjusted P values were calculated using the Benjamini–Hochberg method. Significant differences in the secretion of proteins were called based on the adjusted P value <0.1. Results were visualized using volcano plots using the R package ggplot2. Pathway enrichment analysis of significant differentially secreted proteins identified in our samples and reported to be as neurotoxic factors in other published studies (Didangelos et al., 2016; Guttenplan et al., 2021; Rooney et al., 2021) was performed in R and using the R package enrichR (Xie et al., 2021) v2.1. Significant pathways from the WikiPathways (Martens et al., 2021) were filtered using an adjusted P value <0.05. Enriched genes were visualized using a bubble plot using the R package ggplot2.

The amount of six specific cytokines (IL8, IL6, IL1β, CXCL10, IFNα, and TNFα) was measured in the supernatant by ELISA and Luminex assays (R&D) according to the manufacturer’s instructions. Absorbance of each sample was determined on a spectrophotometer using a Multiskan GO microplate reader (Thermo Fisher Scientific) and normalized to antigen standard curves.

### Quantification and statistical analysis

All statistical analyses were conducted with GraphPad Prism v8.01. In all studies, values are expressed as mean ± SEM or mean ± SD, and all *n* represent biological repeats of independent differentiation experiments if not differently specified in figure legends. Statistical analyses were performed by one-tailed Mann–Whitney *U* test between means of two groups or Dunn’s adjusted Kruskal–Wallis for multiple comparisons, as indicated in the figure legends. Single-cell statistical significance analysis was investigated by the nonparametric Wilcoxon rank sum test. DRIP-qPCR and slot blot were analyzed using two-tailed unpaired Student’s *t* test. Differences were considered statistically significant at *, P < 0.05; **, P < 0.01; ***, P < 0.001; ****, P < 0.0001.

### Data availability

The raw and processed data from the scRNA-seq, bulk RNA-seq, and TP53 sequencing experiments have been deposited in a SuperSeries dataset to the Gene Expression Omnibus archive and can be found using the following Gene Expression Omnibus accession number: GSE193714. Any additional information required to reanalyze the data reported in this article is available from the lead contact upon request.

### Online supplemental material

Fig. S1 shows more details about KO iPSC characterization and neural differentiation. Fig. S2 presents further analysis on iPSC-derived NSCs and neurons. Fig. S3 shows other heatmaps from scRNA-seq of AGS patient–derived astrocytes. Fig. S4 shows AGS patient–derived astrocytes differentiation characterization, GSE56 impacts on IFN score, and p21 gene expression. Fig. S5 shows KO astrocyte Line1 expression levels, RTi impact on IFN scores and transduction, and drugs impact on IFN score, γH2AX, and cC3 expression. Table S1 contains all the Taqman probes used in this work. Table S2 shows all the sequences of the PCR primers used in this work. Table S3 lists all the antibodies used. Table S4 specifies concentrations of all the drugs used in our experiments. Table S5 lists the identified differential expressed genes found in the bulkRNA-seq. Table S6 lists the identified differential expressed genes found in the scRNA-seq. Table S7 contains the list of proteins identified in the MS analysis.

## Supplementary Material

Table S1 contains all the Taqman probes used in this work.

Table S2 shows all the sequences of the PCR primers used in this work.

Table S3 lists all the antibodies used.

Table S4 specifies concentration of all the drugs used in our experiments.

Table S5 lists the identified differential expressed genes found in the bulkRNA-seq.

Table S6 lists the identified differential expressed genes found in the scRNA-seq.

Table S7 lists the proteins identified in the MS analysis.

SourceData F1 contains original blots for Fig. 1.

SourceData F5 contains original blots for Fig. 5.

SourceData FS1 contains original blots for Fig. S1.

SourceData FS4 contains original blots for Fig. S4.
