# Supplementary material for: DNA damage contributes to neurotoxic inflammation in Aicardi-Goutières syndrome astrocytes
Source: J Exp Med. 2022 Mar 9;219(4):e20211121. doi: 10.1084/jem.20211121 (PMC8916121; doi:10.1084/jem.20211121)
Supplement: Table S1 — contains all the Taqman probes used in this work. [file JEM_20211121_TableS1.docx]

TableS1. List of taqman probes

| **Probe** | **Company** | **Code** |
| --- | --- | --- |
| CDKN1A (P21) | Thermo-Fisher | Hs00355782_m1 |
| CXCL8 | Thermo-Fisher | Hs00174103_m1 |
| GAPDH | Thermo-Fisher | Hs02786624_g1 |
| GFAP | Thermo-Fisher | Hs00157674_m1 |
| HPRT1 | Thermo-Fisher | Hs01003267_m1 |
| IFIT1 | Thermo-Fisher | Hs01675197_m1 |
| IL1 beta | Thermo-Fisher | Hs01555410_m1 |
| IRF7 | Thermo-Fisher | Hs01014809_g1 |
| ISG15 | Thermo-Fisher | Hs01921425_s1 |
| SOX2 | Thermo-Fisher | Hs01053049_s1 |
| OCT4 | Thermo-Fisher | Hs03005111_g1 |
| OAS1 | Thermo-Fisher | Hs00973637_m1 |
| P16/CDKN2A | Thermo-Fisher | Hs00923894_m1 |
| PAX6 | Thermo-Fisher | Hs00240871_m1 |
| S100b | Thermo-Fisher | Hs00389217_m1 |
| MB21D1 (cGAS) | Thermo-Fisher | Hs00403553_m1 |
| CARD8 | Thermo-Fisher | Hs01088221_m1 |
| CASP1 | Thermo-Fisher | Hs00354836_m1 |
| RSAD2 | Thermo-Fisher | Hs00369813_m1 |
| IFI27 | Thermo-Fisher | Hs01086373_g1 |
| IL-6 | Thermo-Fisher | Hs00174131_m1 |
| TNFa | Thermo-Fisher | Hs00174128_m1 |
| USP18 | Thermo-Fisher | Hs00276441_m1 |
