# Supplementary material for: DNA damage contributes to neurotoxic inflammation in Aicardi-Goutières syndrome astrocytes
Source: J Exp Med. 2022 Mar 9;219(4):e20211121. doi: 10.1084/jem.20211121 (PMC8916121; doi:10.1084/jem.20211121)
Supplement: Table S2 — shows all the sequences of the PCR primers used in this work. [file JEM_20211121_TableS2.docx]

**TableS2: Sequences of PCR primers**

| **Gene** | **Sequence (5’ 3’)** |
| --- | --- |
| *ACTB* (F) | CGG GGT CTT TGT CTG AGC |
| *ACTB* (R) | CAG TTA GCG CCC AAA GGA C |
| *JUNB* (F) | TGC ACA AGA TGA ACC ACG TG |
| *JUNB* (R) | GCT GAG GTT GGT GTA AAA CGG |
| *HIST1E1E* (F) | CCC ACC GCT CTC AGT AAA AG |
| *HIST1E1E* (R) | ACT CCT CTC CCC GAC TTT GT |
| *TFF1* (F) | TAT GAA TCA CTT CTG CAG TGA G |
| *TFF1* (R) | GAG CGT TAG ATA ACA TTT GCC |
| LINE1 5’UTR (F) | CTCAAGTGGGTCCCTGACTCC |
| LINE1 5’UTR (R) | TCTGTTGGAATACCCTGCCG |
| *GAPDH* (F) | CCACCCATGGCAAAT TCC |
| *GAPDH* (R) | TGGGAT TTCCAT TGATGACAAG |
| *BCL10 (F)* | TTCCTGTGTCGCCTTCTGGG |
| *BCL10 (R)* | TCACAGGTCGGGAGAGGAGG |
| *IL1R1 (F)* | ACCGGCCAGTTGAGTGACAT |
| *IL1R1 (R)* | GTCTTCCCCTAGCACTGGGT |
