# Supplementary material for: DNA damage contributes to neurotoxic inflammation in Aicardi-Goutières syndrome astrocytes
Source: J Exp Med. 2022 Mar 9;219(4):e20211121. doi: 10.1084/jem.20211121 (PMC8916121; doi:10.1084/jem.20211121)
Supplement: Table S3 — lists all the antibodies used. [file JEM_20211121_TableS3.docx]

TableS3. List of antibodies

| **Antibody** | **Company**  **and code** | **Host** | **Dilution** |
| --- | --- | --- | --- |
| Anti β-tubulin III | Babco,  PRB-435P | rabbit | 1/2000 (IF) |
| Anti GAPDH | Abcam, ab9485 | rabbit | 1/2,000 (WB) |
| Anti GFAP | Merck MAB3402 | mouse | 1/2000 (IF)  1/10000 (WB) |
| Anti cC3 | Cell Signaling 9661s | rabbit | 1/200 (IF) |
| Anti 53BP1 | Novusbio 304SS | rabbit | 1/600 (IF) |
| Anti γH2AX | Millipore  05-636 | mouse | 1/200 (IF) |
| Anti EAAT2 | Santa Cruz 365634 | mouse | 1/100 (IF);  1/700 (WB) |
| Anti P-P53ser15 | Cell Signaling 9284s | rabbit | 1/400 (IF);  1/700 (WB) |
| Anti γH2AX | Cell Signaling 9718s | rabbit | 1/400 (IF) |
| Anti TREX1 | Abcam  185228 | rabbit | 1/500 (WB) |
| Anti RNase H2 | Jackson lab  (Reijns et al., 2012) | sheep | 1/1,000 (WB) |
| Anti Actin | Sigma  a2228 | mouse | 1/1000 (WB) |
| Anti Histone H3 | Abcam  185637 | mouse | 1/3000 (WB) |
| Anti RNA/DNA Hybrid S9.6 | Gromak lab | Hybridoma cell line | 1/1000 (SB) |
| Anti ssDNA (clone16-19) | Millipore  MAB3034 | mouse | 1/5000 (SB) |
