# Supplementary material for: DNA damage contributes to neurotoxic inflammation in Aicardi-Goutières syndrome astrocytes
Source: J Exp Med. 2022 Mar 9;219(4):e20211121. doi: 10.1084/jem.20211121 (PMC8916121; doi:10.1084/jem.20211121)
Supplement: Table S4 — specifies concentration of all the drugs used in our experiments. [file JEM_20211121_TableS4.docx]

TableS4. List of drugs

| **Drug** | **Company and code** | **Final Concentration** |
| --- | --- | --- |
| Anakinra | Kineret, Sobi Pharmaceuticals  Syringe 100mg/0.67mL | 1 µg/uL |
| Infliximab | Merck, cat n 170277-31-3 | 20 µg /uL |
| Z-Vad | Invivogen, S7023 | 30 µM |
| MCC950 | Invivogen, inh-mcc | 5 µM |
| H151 | Invivogen,inh-h151 | 5 µM |
| Anti-IL8 | R&D Systems,  MAB208-SP | 2 µg /mL |
| Eculizumab | Soliris, Alexion | 2 µM |
| ATM inhibitor | Selleck Chemicals  S1092 | 10 µM |
| 3TC | Sigma-Aldrich  L1295 | 10 µM |
| D4T | Sigma-Aldrich  D1413 | 1 µM |
| Eculizumab | Commercial name Soliris, produced by Alexion Pharmaceuticals. Kindly provided from leftovers of patients’ doses at the San Raffaele Hospital. | 2 µM |
