# Supplementary material for: DNA damage contributes to neurotoxic inflammation in Aicardi-Goutières syndrome astrocytes
Source: J Exp Med. 2022 Mar 9;219(4):e20211121. doi: 10.1084/jem.20211121 (PMC8916121; doi:10.1084/jem.20211121)
Supplement: SourceData F1 — contains original blots for Fig. 1. [file JEM_20211121_SourceDataF1.pdf]

Figure 1B

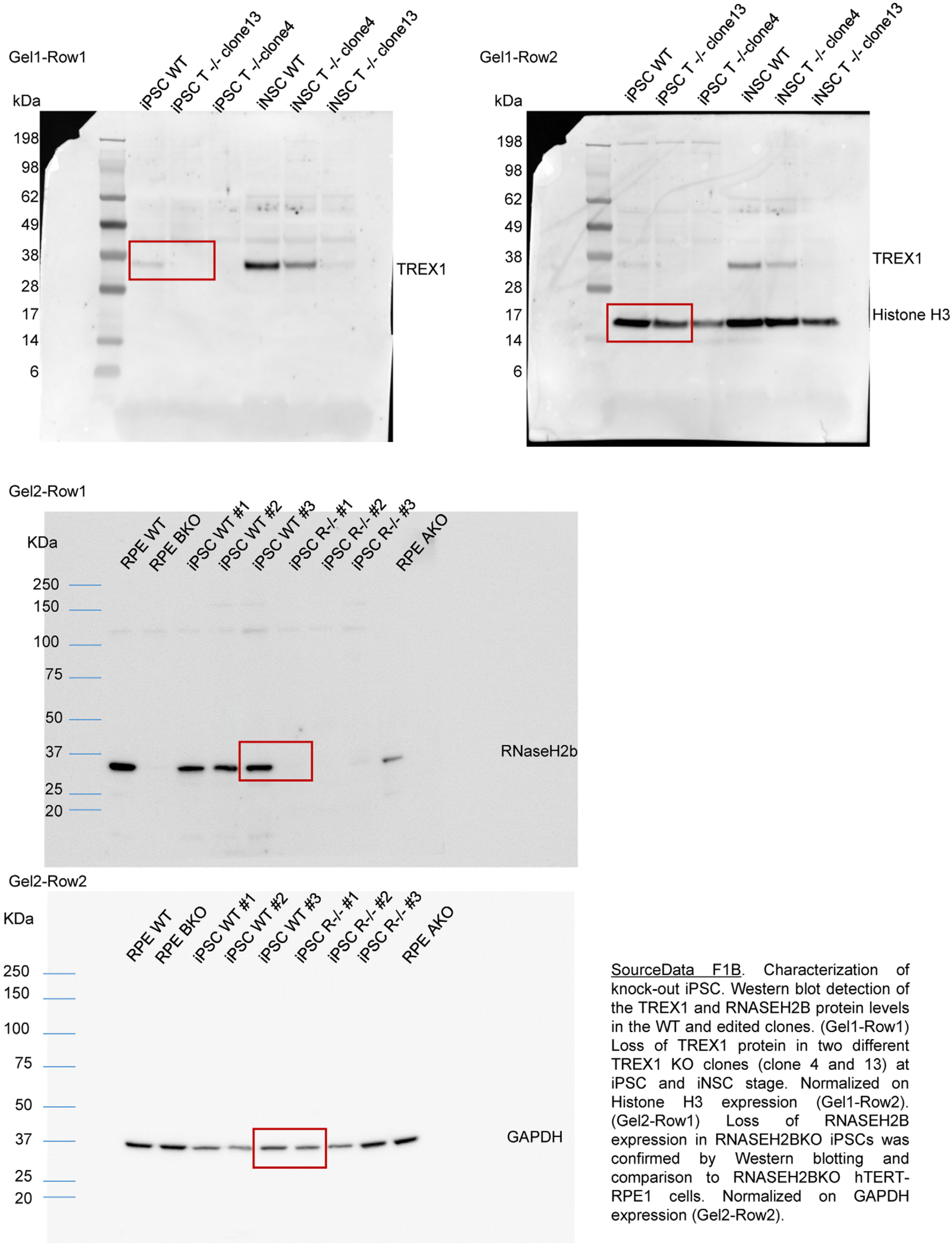

<u>SourceData F1B</u>. Characterization of knock-out iPSC. Western blot detection of the TREX1 and RNASEH2B protein levels in the WT and edited clones. (Gel1-Row1) Loss of TREX1 protein in two different TREX1 KO clones (clone 4 and 13) at iPSC and iNSC stage. Normalized on Histone H3 expression (Gel1-Row2). (Gel2-Row1) Loss of RNASEH2B expression in RNASEH2BKO iPSCs was confirmed by Western blotting and comparison to RNASEH2BKO hTERT-RPE1 cells. Normalized on GAPDH expression (Gel2-Row2).

Figure 1G

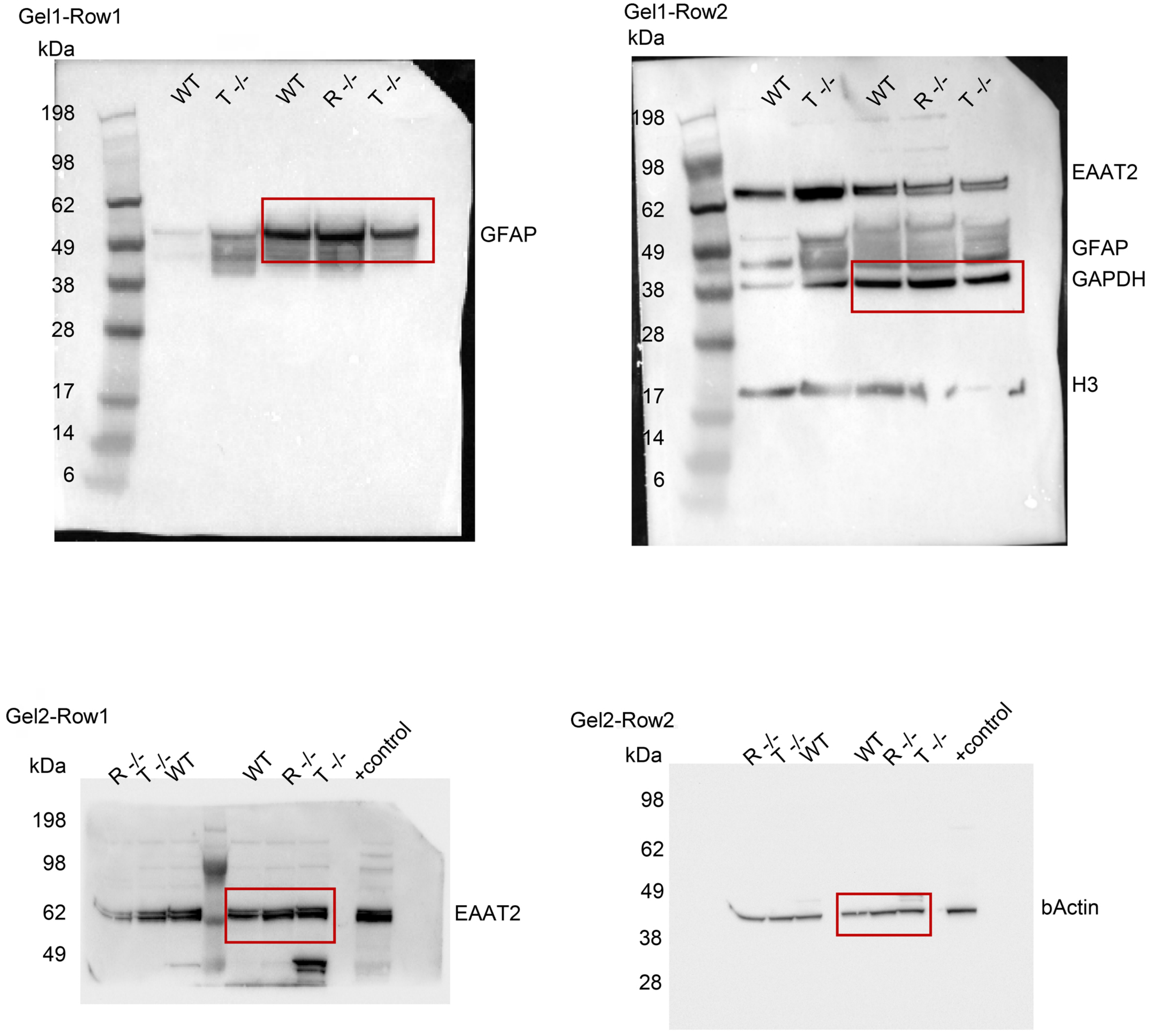

Figure 1. Knock-out induced pluripotent stem cells efficiently differentiate into pro-inflammatory astrocytes. Expression of the astrocyte markers GFAP and EAAT2 by WB at passage 1. Normalized on GAPDH or bActin . Different independently differentiated astrocytes protein lysate were loaded and compared to mixed astroglial population as positive control.
