# Supplementary material for: DNA damage contributes to neurotoxic inflammation in Aicardi-Goutières syndrome astrocytes
Source: J Exp Med. 2022 Mar 9;219(4):e20211121. doi: 10.1084/jem.20211121 (PMC8916121; doi:10.1084/jem.20211121)
Supplement: SourceData F5 — contains original blots for Fig. 5. [file JEM_20211121_SourceDataF5.pdf]

Figure 5A

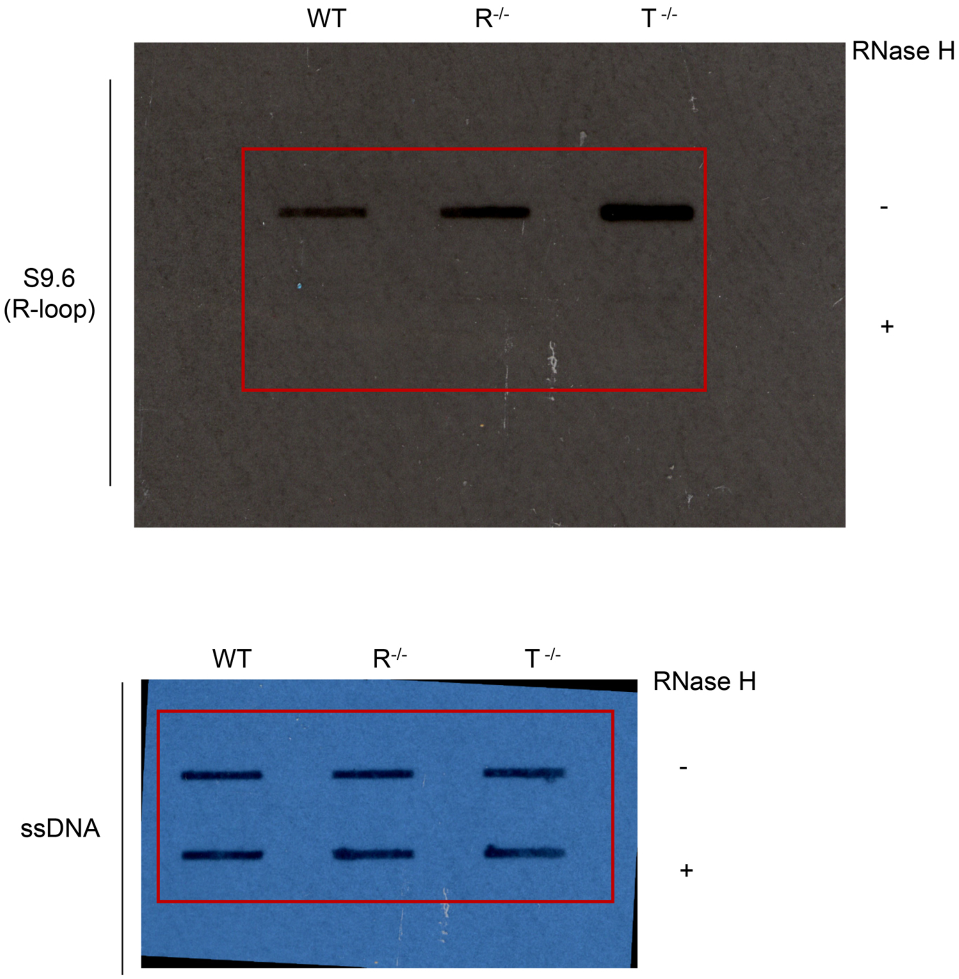

SourceData F5A. AGS knock-out astrocytes accumulate R-loops. Slot blot analysis of genomic DNA extracted from WT, R-/- and T-/- cells treated with the recombinant RNase H (RNH) enzyme for 2.5 h at 37 °C and probed with S9.6 and ssDNA antibodies. Slot blot probed with the antibody recognizing ssDNA was used as a loading control.
