# Supplementary material for: DNA damage contributes to neurotoxic inflammation in Aicardi-Goutières syndrome astrocytes
Source: J Exp Med. 2022 Mar 9;219(4):e20211121. doi: 10.1084/jem.20211121 (PMC8916121; doi:10.1084/jem.20211121)
Supplement: SourceData FS1 — contains original blots for Fig. S1. [file JEM_20211121_SourceDataFS1.pdf]

Figure S1A

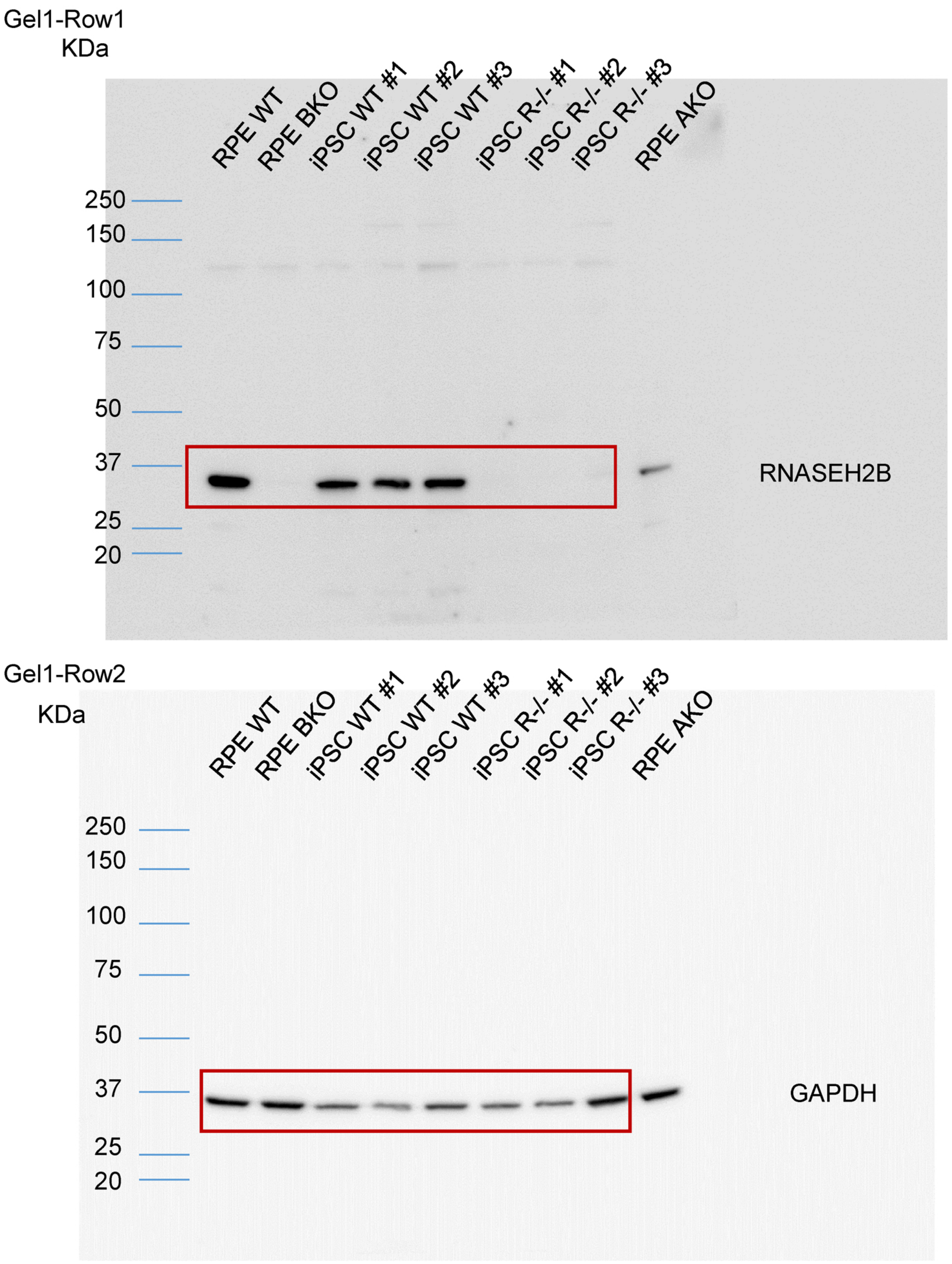

SourceData F1B. Characterization of knock-out iPSC. Western blot detection of RNASEH2B protein levels in the WT and edited clones. (Gel1-Row1) Loss of RNASEH2B expression in RNASEH2BKO iPSCs was confirmed by Western blotting and comparison to RNASEH2BKO hTERT-RPE1 cells. Normalized on GAPDH expression (Gel1-Row2). Different independently differentiated astrocytes protein lysate were loaded.
