# Supplementary material for: DNA damage contributes to neurotoxic inflammation in Aicardi-Goutières syndrome astrocytes
Source: J Exp Med. 2022 Mar 9;219(4):e20211121. doi: 10.1084/jem.20211121 (PMC8916121; doi:10.1084/jem.20211121)
Supplement: SourceData FS4 — contains original blots for Fig. S4. [file JEM_20211121_SourceDataFS4.pdf]

Figure S4

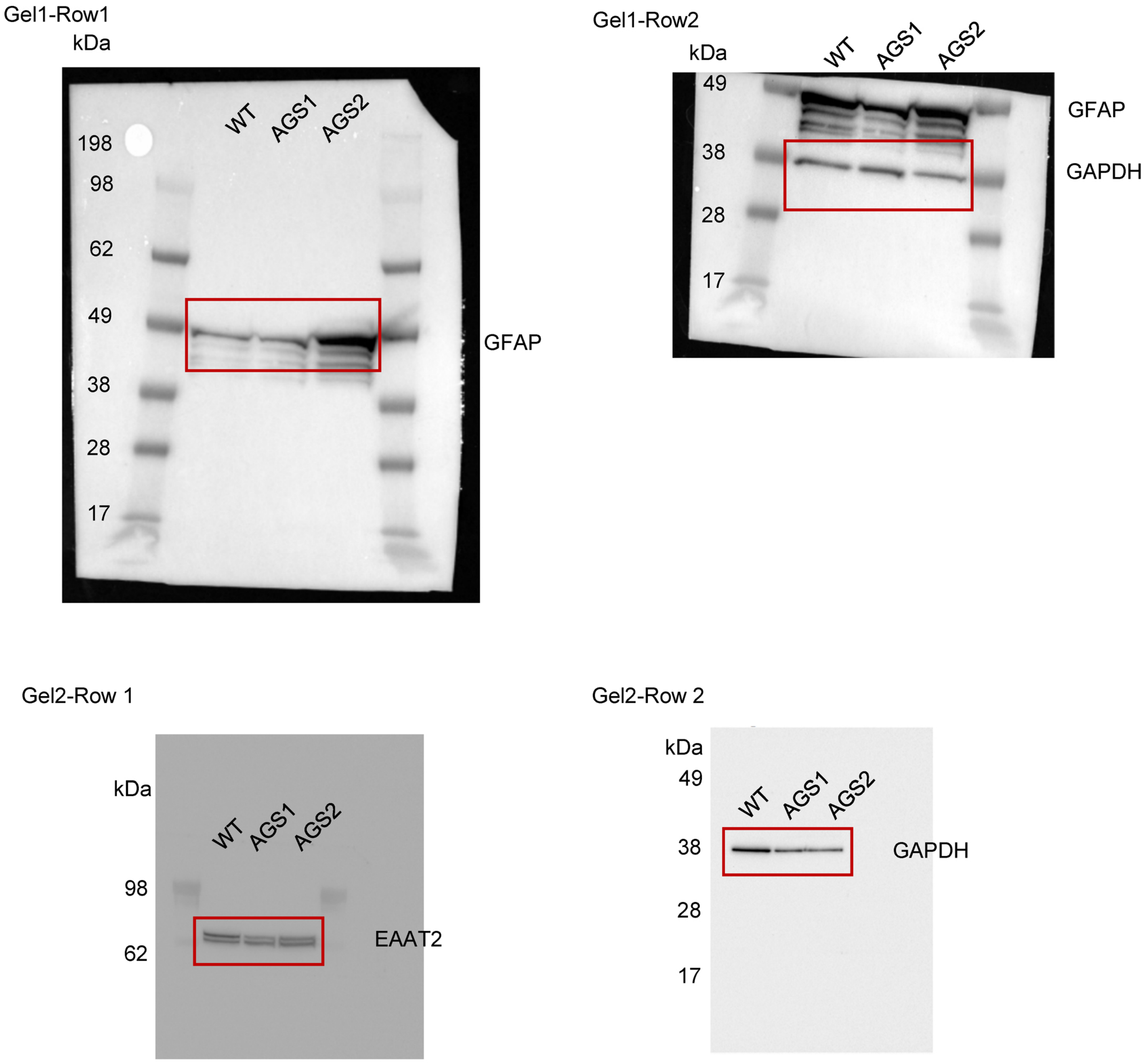

Figure S4. AGS patient-derived efficiently differentiate into pro-inflammatory astrocytes Expression of the astrocyte markers GFAP (Gel1-Row1) and EAAT2 (Gel2-Row1) by WB normalized on GADPH (Gel1-Row2 and Gel2-Row2) levels of iPSC-derived proinflammatory astrocytes at passage 1.
